# Nonpharmacological Multimodal Interventions for Cognitive Functions in Older Adults With Mild Cognitive Impairment: Scoping Review

**DOI:** 10.2196/70291

**Published:** 2025-05-12

**Authors:** Raffy Chi-Fung Chan, Joson Hao-Shen Zhou, Yuan Cao, Kenneth Lo, Peter Hiu-Fung Ng, David Ho-Keung Shum, Arnold Yu-Lok Wong

**Affiliations:** 1 Department of Rehabilitation Sciences Hong Kong Polytechnic University Hong Kong China (Hong Kong); 2 Department of Social Work and Social Administration University of Hong Kong Hong Kong China (Hong Kong); 3 Department of Food Science and Nutrition Hong Kong Polytechnic University Hong Kong China (Hong Kong); 4 Research Institute for Smart Ageing Hong Kong Polytechnic University Hong Kong China (Hong Kong)

**Keywords:** mild cognitive impairment, multimodal intervention, prevention, randomized controlled trial, cognitive decline

## Abstract

**Background:**

As the global population ages, the prevalence of dementia is expected to rise significantly. To alleviate the burden on health care systems and the economy, it is essential to develop effective strategies to enhance cognitive function in older adults. Previous studies have shown that combined nonpharmacological interventions can improve cognition across various domains in older individuals. However, there is no established gold standard for the exact combination and duration of these interventions, which makes it challenging to assess their overall effectiveness.

**Objective:**

Given the diversity of nonpharmacological multimodal interventions aimed at preventing cognitive decline in older adults with mild cognitive impairment (MCI), this scoping review sought to identify and summarize the characteristics and outcomes of these interventions.

**Methods:**

We adhered to the Arksey and O’Malley methodological framework and the PRISMA-ScR (Preferred Reporting Items for Systematic Reviews and Meta-Analyses Extension for Scoping Reviews) and searched 4 electronic databases (MEDLINE, PsycINFO, CINAHL, and Web of Science) systematically on July 6, 2023, and updated the search on April 17, 2024, using specific terms and keywords.

**Results:**

This review included 45 studies from 18 countries with 4705 participants from 2014 to 2024 encompassing different combinations of physical training (PT), cognitive training (CT), nutrition intervention, psychosocial intervention, social activities, and electrical stimulation. There is a growing numbers of studies combining PT and CT for MCI treatment, with additional modalities often added to address various aspects of the condition. Compared to single-modal interventions and usual care, multimodal approaches demonstrated significantly better improvements in cognition domains such as attention, global cognition, executive function, memory, processing speed, and verbal fluency. Technology has been instrumental in delivering these interventions and enhancing the effects of PT and CT. Multimodal interventions also show promise in terms of acceptability and user experience, which can improve treatment adherence.

**Conclusions:**

Research is limited regarding the cost-effectiveness and optimal dosage of these interventions, making it difficult to assess the additional benefits of incorporating more modalities. Future research should examine the long-term effects of incorporating multiple modalities, using standardized MCI criteria and outcome measures.

## Introduction

### Overview

Mild cognitive impairment (MCI) represents a transitional stage between normal aging and dementia, characterized by cognitive decline greater than expected for age, impacting one or more domains such as attention, memory, orientation, executive functioning, language, and visuospatial skills [1,2]. However, the decline does not significantly interfere with daily activities [3]. The lifetime prevalence of MCI in individuals aged above 60 years is estimated to be 15% to 20% [4], with this prevalence increasing with age. The annual transition rate from MCI to various subtypes of dementia ranges from 10% to 15% and can reach up to 25.2% for adults aged 80 to 84 years [5,6]. Older adults with MCI are 46% more likely to develop dementia within 3 years compared to just 3% in the normal-aging population [7].

The trajectory for individuals with MCI may vary, leading to dementia, stable cognition, or a return to normal cognition function [6]. Untreated MCI may progress to dementia, a neurodegenerative disease that significantly impacts daily functions and affects one’s physical, psychological, social, and economic aspects. Dementia also directly affects one’s caregivers, families, and society [8]. With the global population of individuals aged 60 years and above projected to double from 1 billion in 2020 to 2 billion by 2050 [9], there is mounting concern regarding the rising prevalence of dementia and the urgent need for preventive measures to address the associated social and economic burdens. Factors, such as modifiable risk factors, genetics, and interventions, can affect MCI progression [10]. Therefore, understanding various interventions and their effectiveness in preventing progression from MCI to dementia is crucial given the rising prevalence of dementia among older adults.

Currently, there is no gold standard for treatments or interventions to manage MCI. The American Academy of Neurology guidelines indicate insufficient empirical evidence to support pharmacological treatments for MCI in older adults [11]. Conversely, numerous studies have advocated nonpharmacological interventions, such as physical training (PT) or cognitive training (CT), as effective strategies for managing MCI [11-13].

### Nonpharmacological Interventions

PT, including aerobic, strengthening, and balance exercises, has been shown to stimulate norepinephrine release in the brain, promote brain plasticity, increase brain volume, and enhance cerebral blood flow [14]. These effects are crucial for improving cognition, mood, and physiological abilities [15]. Combining aerobic and strengthening exercises has been identified as particularly effective for cognitive improvement [16].

On the contrary, CT primarily uses cognitive stimulation and repetitive tasks to enhance various cognitive domains, particularly memory, attention, and executive function [17]. The effectiveness of CT in improving cognition lies in its capacity to strengthen the functioning and plasticity of neural networks and cognitive reserves [17]. Research suggested that memory-focused CT increased activation and connectivity in the frontal, temporal, and occipital brain regions [18]. These areas are crucial for memory, motor function, processing, attention, language, mood, and problem-solving. Therefore, CT shows promise for improving overall cognition in older adults with MCI.

Given that both PT and CT can enhance brain plasticity and stimulate brain regions responsible for various cognitive functions, their combined application, whether delivered separately or through dual tasking, is the most common approach for managing cognitive impairment in MCI. This multifaceted approach effectively targets different aspects of cognitive decline, countering cognitive decay and neurodegeneration.

Growing evidence suggests that engaging in more social activities (SA) can lower the risk of cognitive decline in individuals with MCI [5], making SA a potential key modality for MCI management. SA involves participation in activities that allow interactions or engagements with others [5]. Karp et al [19] found that older adults with MCI who participated in a broader range of activities, including mental stimulation, physical activities, and social recreation, had a lower risk of developing dementia than those who participated in fewer or no such activities. On the other hand, beyond PT, CT, and SA, emerging evidence suggests that modifiable lifestyle factors, including diet and nutritional intervention (NI), electrical stimulation, or psychosocial intervention (PI) may also improve cognitive functions in this population [10,20].

### Multimodal Interventions

A multimodal intervention, integrating various methods such as PT, CT, SA, NI, electrical stimulation, and PI, either sequentially or simultaneously, addresses cognitive decline across different domains of MCI. This approach has been proven effective in managing MCI in older adults, enhancing cognitive abilities, mood, sleep, activities of daily living, functional capacities, and physical abilities, with benefits lasting up to 2 years [14]. A systematic review and meta-analysis [21] showed that combining 2 or more interventions had small to medium effects on global cognition, memory, executive function, and verbal fluency, demonstrating a synergistic effect. Studies also showed that multimodal interventions outperformed single-modal interventions in managing MCI [15,16]. Another systematic review and meta-analysis [22] also supported that combined PT and CT had a small to medium effect on global cognition than various types of cognitive-only interventions in older adults with MCI. However, these reviews solely focused on the effects of combined PT and CT [22] or compared the effectiveness of multimodal interventions only with single-modal interventions [21], thus lacking direct comparisons between various types of multimodal interventions to inform researchers or clinicians regarding which combinations of multimodal interventions would yield better results. In addition, previous reviews did not consider the user experience of multimodal interventions, which limits the clinical applicability of their findings and important factors to determine the feasibility of multimodal interventions. Thus, a comprehensive evaluation of multimodal interventions should include both feasibility and user experience to optimize benefits.

### Optimizing Multimodal Interventions: Technology, Dosage, and Cost-Effectiveness

The increasing adoption of digital health care technology in managing MCI [23] could enhance the delivery and reduce the costs of interventions, especially benefiting those in remote areas [24]. Given the high prevalence and economic burden of MCI on communities and health care systems, technology such as the use of computerized CT (CCT) can help mitigate costs associated with nonpharmacological interventions, addressing the shortage of trained professionals. While previous systematic reviews and meta-analyses have acknowledged the role of technology in MCI management, they mainly focused on CCT [24-26] without considering the broader potential of technology beyond CCT.

Addressing the impact of different dosages of nonpharmacological interventions for MCI is crucial for developing effective and sustainable therapeutic strategies. This involves determining the optimal frequency, intensity, type, and duration of interventions to maximize cognitive benefits while balancing time and financial costs [27]. A study [27] highlighted that CT, PT, NI, and the majority of combined PT and CT significantly improved cognition in individuals with MCI, with particularly effective doses being 1 to 2 sessions per week with 60 to 120 minutes per session and interventions lasting over 12 weeks. However, as this study focused on only one type of multimodal intervention (combined PT and CT), the dosage effects of other nonpharmacological multimodal interventions remain uncertain. Therefore, this scoping review aimed to summarize dosage effects from the literature on multimodal interventions.

In addition, managing cognitive decline imposes a significant global economic burden. The cost-effectiveness of multimodal interventions varies by region because of the differences in health care resources. Although previous research supported the effectiveness of multimodal interventions for cognitive decline, their adoption of multiple modalities often increases cost. However, no prior review has summarized the cost-effectiveness of these interventions, so this scoping review aimed to address this important gap to inform clinical decision-making.

### Rationale for a Scoping Review

Considering the variety of multimodal MCI interventions, both with and without technology, and the diverse methodologies and research focus of the existing literature, a scoping review is warranted to identify the current research gaps to inform future research and clinical practice. The complexity and heterogeneity of the interventions, coupled with the rapidly evolving nature of this field, make a systematic review less feasible for comprehensively mapping the use of different types of multimodal interventions and investigating the current research trends. As such, this scoping review aimed to map and describe the latest development in MCI interventions and provide researchers and clinicians with insights into current trends and limitations of the existing approaches and studies. Specifically, this review mapped the current landscape of nonpharmacological multimodal interventions for older adults with MCI, identified and summarized the components of these interventions, research trends, and the use of different outcome measures. It aimed to enhance the understanding of MCI management and provide future research directions on multimodal interventions.

## Methods

### Overview

This review adhered to the Arksey and O’Malley methodological framework [28] and the PRISMA-ScR (Preferred Reporting Items for Systematic Reviews and Meta-Analyses Extension for Scoping Reviews) [29]. The protocol was registered on the Open Science Framework platform [30]. The review process involved five key steps: (1) formulating the research questions; (2) devising the search strategy; (3) identifying and selecting relevant studies; (4) data charting; and (5) synthesizing and presenting findings.

### Identified Research Questions

This review explored the following research questions:

1. What are the research trends in multimodal interventions for older adults with MCI?
2. What components were included in these multimodal interventions? What results have been reported?
3. How cost-effective were the identified multimodal interventions?
4. What role did technology play in these interventions for older adults with MCI?
5. What insights were available regarding the acceptability, user experiences, and dose responses of these interventions?

### Identifying and Selecting Relevant Studies

We conducted a search across 4 databases—MEDLINE, PsycINFO, CINAHL, and Web of Science—on July 6, 2023, and updated the search on April 17, 2024, using specific Medical Subject Headings terms and keywords such as “combine,” “multi,” “dual,” “mix interventions, and “mild cognitive impairment” (Multimedia Appendix 1). Although quality assessment is optional for scoping reviews [31], this review included only randomized controlled trials to enhance study quality. Two independent reviewers (JHSZ and RCFC) screened titles, abstracts, and full texts against the eligibility criteria (Textbox 1). Disagreements were resolved by consensus or by consulting a third reviewer (AYLW). The interrater reliability of the screening process as measured by the kappa coefficient was 0.87.

**Selection criteria for the scoping review.**

**Inclusion criteria**

- Participants: diagnosed with mild cognitive impairment (MCI) by clinicians or psychologists using well-established criteria
- Intervention: at least one combination of a nonpharmacological multimodal intervention in managing older adults with MCI
- Control: received at least one or multiple forms of an intervention, a placebo or sham training, health education, or treatment as usual
- Outcome: must use at least one well-established measurement for testing cognitive outcomes
- Study design: must be an experimental study (randomized controlled trial or quasi-experimental study)
- Other: full-text and peer-reviewed study written in English

**Exclusion criteria**

- Participants: diagnosed with dementia or the cognitive impairment resulted from drug use or psychiatric or other neurological disorders (eg, bipolar disorder, schizophrenia, stroke, Parkinson disease, or epilepsy)
- Intervention: pharmacological interventions or nonpharmacological experimental studies with a single-modal intervention
- Study design: systematic reviews, scoping reviews, opinion letters, conference proceedings, dissertations, and research design protocols
- Other: gray literature

### Definition of Types of Intervention and Control Groups

The included studies featured diverse intervention components and control groups. Detailed operational definitions for these interventions and control groups are provided in Textbox 2.

**Operational definition of the intervention and control groups in the included studies.**

**Interventions**

- Physical training: activities, exercises, or training that required older adults to do physical activities with or without the guidance or supervision of a professional trainer or clinician
- Cognitive training: activities or training that used a standardized systematic cognitive stimulation, rehabilitation task, and training to improve cognitive function
- Nutrition intervention: the use of any type of dietary supplement, including herb extract, or any form of dietary counseling
- Psychosocial intervention: the use of activities, training, counseling, therapy, or education that aimed to improve psychological well-being, including music therapy and mindfulness
- Social activities: activities that encourage social engagement or facilitate social interaction between older adults
- Electric current stimulation: the use of current stimulation including transcranial alternating current stimulation and transcranial direct current stimulation

**Control groups**

- Active control: a group of participants who received at least one form of intervention
- Placebo control: a group of participants who received a placebo or sham training
- Health education control: a group of participants who received health education
- Inactive control: a group of participants who received no additional treatment, treatment as usual, or only health advice

### Data Extraction

Relevant data, including authors, publication year, country, place of recruitment, diagnostic criteria, participants, intervention types, outcomes, treatment frequency and duration, follow-up time points, use of measurement tools, control group characteristics, results, and interpretations of findings, were extracted by 2 independent reviewers (JHSZ and RCFC). All extracted findings were compared. Discrepancies were reconciled by consensus or through consultation with a third reviewer (AYLW).

## Results

### Study Selection and Characteristics

**Figure 1:**
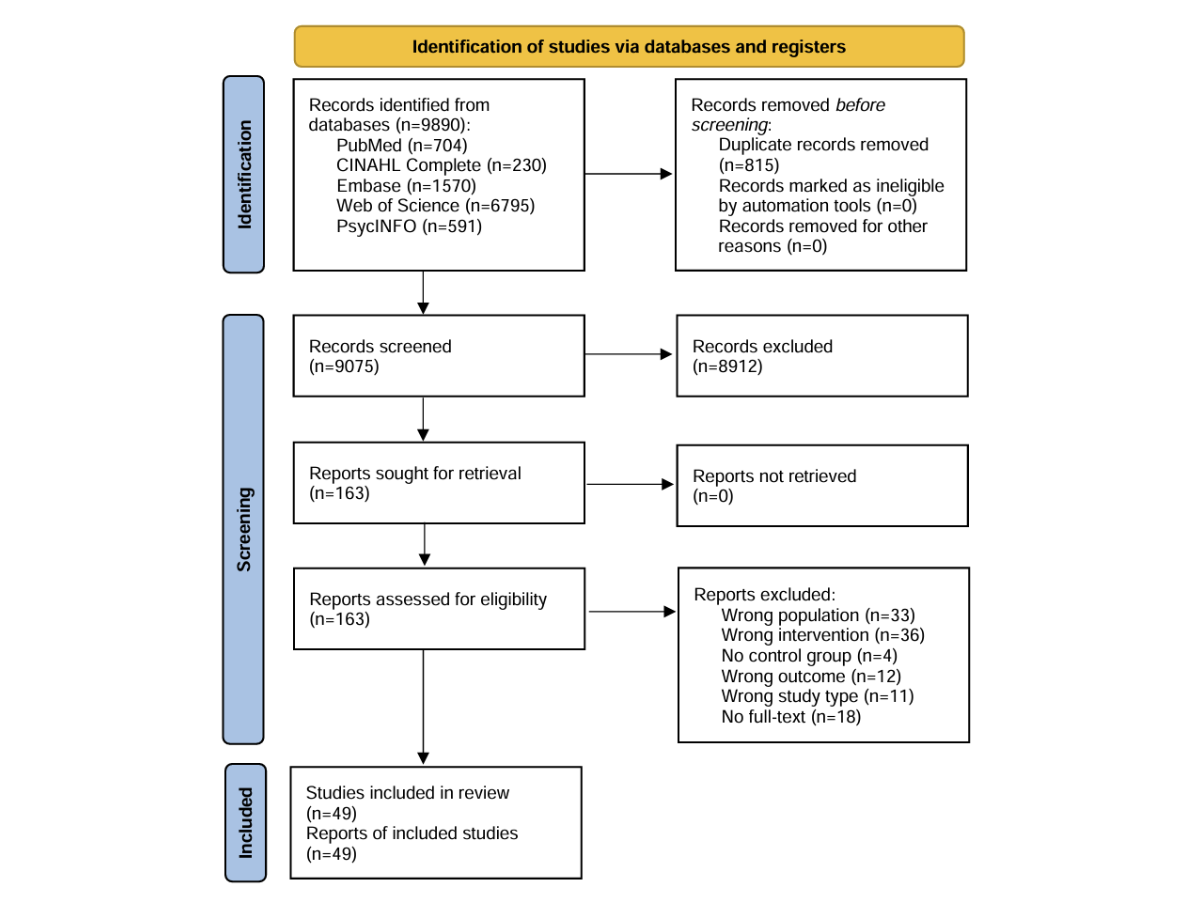
PRISMA (Preferred Reporting Items for Systematic Reviews and Meta-Analyses) 2020 flow diagram of literature search and screening.

The initial search identified 9890 articles. After removing 815 duplicates, 9075 titles and abstracts were screened; 163 out of 9075 titles and abstracts were selected for full-text screening. Studies based on the same cohort were counted as a single study, including 4 studies by Hagovská and Nagyova [32], Hagovská and Olekszyova [33,34], and Hagovská et al [35] and 2 studies by Liao et al [36,37]. Exclusions were made for reasons including incorrect target population (n=33), absence of multimodal intervention (n=36), lack of a control group (n=4), different study outcomes (n=12), absence of cognitive outcomes (n=11), and unavailability of full text (n=18). Ultimately, 45 studies from 49 articles published between 2014 and 2024, encompassing a total of 4705 participants, were included in this review. A detailed description of the study selection is presented in Figure 1.

The characteristics of the included studies, including participant characteristics, intervention tasks, treatment duration and frequency, and assessment time points, are presented in Multimedia Appendix 2. These studies used various criteria to diagnose MCI, such as the Peterson criteria (n=14) [15,16,38-49], the Albert criteria (n=5) [50-54], and cutoff scores from established screening tools like the Alzheimer Disease Assessment Scale–Cognitive subscale (cutoff score not provided) [38,40], Mini-Mental State Examination between 20 and 27 [55], Montreal Cognitive Assessment with scores up to 28 [15,23,36,37,52,56-65], Clinical Dementia Rating scores from 0.5 to less than 1 [45,60,66], or direct diagnoses from professional psychologists. Table 1 provides a detailed description of the diagnostic criteria used for a comprehensive reference.

**Table 1:** Diagnostic criteria used in the included studies.

Criteria	Definition
Peterson criteria [11]	Self-report cognitive decline Objective cognitive impairment compared with age No impact on daily functioning No dementia
Albert criteria [3]	Cognitive change reported by the patient, informant, or clinician Objective evidence of cognitive impairment in one or more domains (typically 1 to 1.5 SDs below the mean when compared with their peers with matched age and education level) Independent in functional abilities No dementia

### Publication or Study Trend

**Figure 2:**
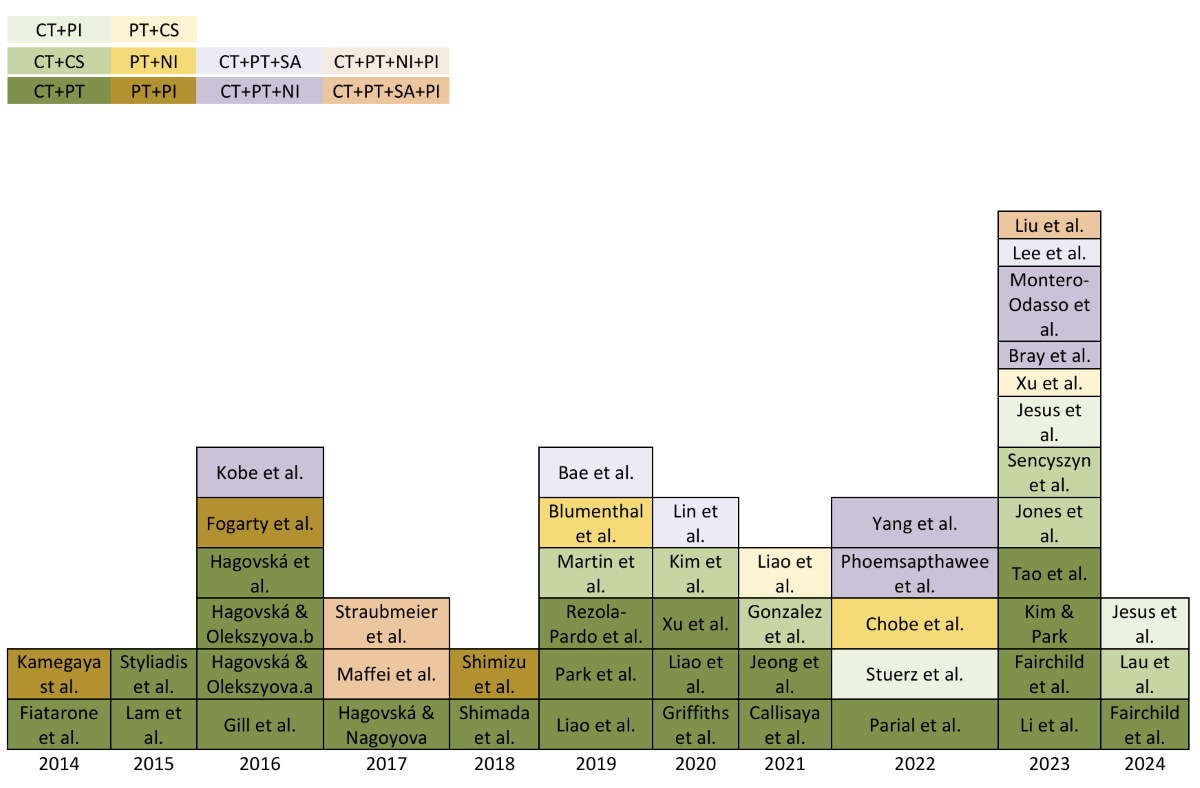
The number of studies that incorporated multimodal interventions for mild cognitive impairment throughout the years. CS: current stimulation; CT: cognitive training; NI: nutritional intervention; PI: psychosocial intervention; PT: physical training; SA: social activities.

There has been a noticeable increase in both the number of studies and the variety of multimodal intervention combinations from 2014 to 2024 (Figure 2 [15,16,32-78]). Specifically, studies incorporating NIs as part of multimodal MCI interventions grew from 1 in 2016 to 3 in both 2020 and 2023. In addition, the use of technology in delivering multimodal interventions has gradually increased from 1 study in 2015 to 5 in 2023.

### Types of Multimodal Interventions

The included studies used combinations of 2, 3, and 4 types of interventions. Although most included studies primarily focused on combining PT and CT, there is an increasing number of studies integrating additional interventions to address the multifaceted nature of MCI. The following section details these specific combinations, the types of control groups used, and the overall outcomes across cognitive domains, such as attention, executive function, global cognition, memory, processing speed, and verbal fluency. Comprehensive outcomes, measurement tools, and targeted cognitive domains of the included studies are summarized in the following sections.

### Bimodal Interventions

#### Overview

Most studies that used combined PT and current stimulation [45,67], or PT and NI [56,58] reported significantly greater improvements in various cognition domains compared to active control groups, while the use of combined CT and PI [61,68,69] reported significantly greater improvements compared to inactive control groups. However, studies with combined PT and CT showed mixed results. Conversely, most studies comparing combined PT and PI [39,41,48], CT and electroacupuncture [42], or CT and current stimulation [44,47,53,60,62] with an active or inactive control intervention found little to no significant advantage in the intervention groups (Table 2).

**Table 2:** Studies that incorporated a bimodal intervention and outcomes (n=35).

Study		Sample size (control or controls sample size)	Control or controls	Measured cognitive domains	Clinical outcomes
**PT^a^+CT^b^**					
	Callisaya et al [57], 2021	44 (49)	IN^c^	GC^d^ (COWAT^e^, DSCT^f^, HVLT^g^, TMT-A^h^, TMT-B^i^, and SCWT^j^)	No significant difference between the two groups for all tests (*P*>.05)
	Fairchild et al [51], 2024	36 (36)	IN	EF^k^ (DST^l^, TMT-B, and SCWT) ME^m^ (WMS-LM I^n^ and II^o^, and RAVLT^p^) PS^q^ (TMT-A and SCWT)	No significant difference between the two groups for all tests (*P*>.05)
	Fiatarone Singh et al [38], 2014	27 (22; 24; 27)	CT only PT only PC^r^	ATT^s^ (SDMT^t^) GC^u^ (ADAS-Cog^v^) EF (Matrices and Similarities subtests of the WAIS-III^w^) ME (ADAS-Cog, BVRT^x^, and LM^y^ from WMS-III^z^) PS (SDMT) VF^aa^ (COWAT and animal naming)	The intervention group did not show significant differences compared to other groups across all tests (*P*>.05)
	Gill et al [59], 2016	23 (21)	PT only	GC (composite score) EF (TMT-A and TMT-B) ME (AVLT^ab^) PS (DSST^ac^) VF (VFT^ad^)	GC (mean difference 0.2, *P*=.04^ae^) EF (mean difference 0.11, *P*=.60) ME (mean difference 0.3, *P*=.02) PS (mean difference −0.06, *P*=.78) VF (mean difference 0.62, *P*=.003)
	Griffiths et al [52], 2020	35 (35)	IN	ATT (TMT-A and TMT-B) EF (BD^af^ of the WAIS-IV^ag^) ME (DST-F^ah^, DST-B^ai^, DSS^aj^ from WAIS-IV, VFT, and WLL^ak^)	ATT (TMT-A: mean difference −27.86, *P*=.36) EF (mean difference 0.34, *P*=.58) ME (DST-F and DST-B: mean difference 1.23, *P*=.60; DSS: mean difference 1.030, *P*=.31; VFT-Letter^al^: mean difference 4.59, *P*=.001; VFT-Category^am^: mean difference 2.81, *P*=.23)
	Hagovská et al [35], 2016	40 (40)	PT only	ATT (SCWT) GC (ACE^an^ and MMSE^ao^) ME (ACE and AVLT) PS (DRT-II^ap^) VF (ACE)	ATT (SCWT: η2=0.0001, *P*=.97) GC (ACE: Cohen d=0.71, *P*=.002; MMSE: η2=0.189, *P*=.001) ME (ACE: Cohen d=0.64, *P*=.007; AVLT: η2=0.173, *P*=.001) PS (DRT-II: η2=0.033, *P*=.11) VF (Cohen d=0.73, *P*=.001)
	Jeong et al [40], 2021	13 (13)	HE^aq^	ATT (TMT-A and TMT-B) GC (ADAS-Cog and KMMSE^ar^) PS (DSST)	Group×time interaction ATT—TMT-A: *P*<.05; TMT-B: *P*=.01 GC (ADAS-Cog: *P*=.11); KMMSE (*P*=.72) PS (*P*=.02)
	Kim and Park [63], 2023	21 (21)	CT only	EF (EFPT-K^as^ and FAB^at^)	EF (EFPT-K: η2=0.132, *P*<.01; FAB: η2=0.305, *P*<.001)
	Lam et al [74], 2015	93 (115; 114; 101)	CT only PT only SA only	ATT (VFT-Category) GC (ADAS-Cog, CDR-SOB^au^, and CMMSE^av^) ME (list learning delayed recall test)	ATT (VFT-C: χ2=23.38, *P*<.001) GC (ADAS-Cog: χ2=3.31, *P*=.2; CDR-SOB: χ2=1.82, *P*=.61; CMMSE: χ2=4.28, *P*=.23) ME (χ2=3.31, *P*=.35)
	Li et al [66], 2023	19 (35; 30)	CT only IN	ATT, EF, ME, language, and spatial ability (AVLT, STT-A^aw^ and STT-B^ax^, CFT^ay^, SCWT, and BNT-30^az^) GC (ADAS-Cog and CMMSE)	ATT, EF, and ME: (AVLT immediate recall: mean difference 0.46, *P*>.05; STT-A: mean difference −0.28, *P*>.05; STT-B: mean difference −0.15, *P*>.05; CFT: mean difference 0.34, *P*>.05; BNT-30: mean difference −1.14, *P*>.05) GC: ADAS-Cog (mean difference 0.03, *P*>.05; MMSE: mean difference 0.22, *P*>.05)
	Liao et al [36,37], 2019, 2020	16 (18)	PT and CT	GC (MoCA^ba^) EF (EXIT-25^bb^ and SCWT) ME (CVVLT^bc^)	Group×time interaction GC (*P*=.18) EF (EXIT-25: *P*=.72; SCWT: number: *P*=.84; time: *P*=.32) ME (CVVLT: immediate recall: *P*=.15; delayed recall: *P*=.12)
	Parial et al [15], 2022	25 (26)	HE	GC (MoCA) EF (TMT-B) ME (DST)	GC (Wald χ2=26.88, *P*<.001) EF (Wald χ2=18.67, *P*<.001) ME (immediate recall: Wald χ2=16.97, *P*<.001; delayed recall: Wald χ2=11.89, *P*<.003)
	Park et al [72], 2019	25 (24)	IN	GC (ADAS-Cog) EF (DSST) ME (DST)	GC: ADAS-Cog—*P*<.01; 95% CI −1.9 (−0.9 to −2.3) EF: DSST—*P*<.01; 95% CI −6.3 (−4.2 to −8.4) ME: DST—*P*=.02; 95% CI −0.6 (−0.3 to 1.6)
	Rezola-Pardo et al [70], 2019	43 (42)	PT only	Overall cognitive performance (MoCA, symbol search and coding test from WAIS-IV, semantic fluency test, VFT, RAVLT, and TMT-A)	MoCA (Cohen d=0.15; *P*=.90) Symbol search (Cohen d=0.06; *P*=.85) Semantic fluency (Cohen d=0.01; *P*=.28) VFT (Cohen d=0.02; *P*=.67) RAVLT (Cohen d=0.11; *P*=.70) TMT-A (Cohen d=0.04; *P*=.65)
	Shimada et al [71], 2018	137 (129)	HE	GC (MMSE) EF (TMT-A and TMT-B) ME (WMS-LM II and RAVLT) VF (VFT)	CG (mean difference 0.8, *P*=.01) EF (mean difference −0.4, *P*=.35) ME (WMS-LM II: mean difference 1.0, *P*=.004; RAVLT: mean difference 0.2, *P*=.35) VF: (mean difference 2.2, *P*=.002)
	Styliadis et al [16], 2015	14 (14; 14; 14; 14)	PT only CT only HE IN	GC (MMSE)	The intervention group did not show significant differences compared to other groups across all tests (*P*>.05)
	Tao et al [73], 2023	51 (52)	IN	GC (MoCA and MMSE)	GC (MoCA: η2=0.442, *P*=.001; F=39.550; MMSE: η2=0.33, *P*<.001; F=24.614)
	Xu et al [65], 2020	5 (6; 6)	NI only IN	GC (HK-MoCA^bd^)	GC: improvement only in the NI^be^-only group (*P*=.008)
**PT+PI^bf^**					
	Fogarty et al [39], 2016	22 (18)	PI only	ATT (TEA^bg^) ME (RBMT^bh^) PS (digit symbol test)	ATT: TEA (visual selective ATT: Cohen d=0.15, *P*=.82; F2,70=0.196; sustained ATT: Cohen d=0.127, *P*=.88; F2,62=0.125; ATT switching: Cohen d=0.0487, *P*=.13; F2,70=2.071) ME (Cohen d=0.583, *P*=.06; F2,70=2.972) PS (Cohen d=0.16, *P*=.80; F2,72=0.230)
	Kamegaya et al [41], 2014	19 (24)	IN	GC (Five-Cog test) EF (DSST and YKSSTbi)	GC (F=2.999, *P*=.09; F1,38=2.999) EF (DSST: F=1.165, *P*=.29; YKSST: F=0.096, *P*=.76; F1,38=1.165)
	Shimizu et al [48], 2018	30 (9)	PT only	GC (FAB)	GC: effect size (r=0.002, *P*=.81)
**PT+NI**					
	Blumenthal et al [56], 2019	40 (41; 41; 38)	PT only NI only HE	GC (modified CDR-SOB) EF (TMT-A, TMT-B, SCWT, DST-F, DST-B, DSST, Ruff 2 and 7 Test, and animal naming) ME (HVLT-revised and CFT) VF (COWAT and animal naming)	GC (Cohen d=0.36, *P*=.03) EF (Cohen d=0.4, *P*=.01) ME (PT factor: Cohen d=0.19, *P*=.24; NI factor: Cohen d=0.15, *P*=.35) VF (PT factor: Cohen d=0.12, *P*=.45; NI factor: Cohen d=0.23, *P*=.24)
	Chobe et al [58], 2022	24 (25; 23)	PT only NI only	ATT (DST-F and DST-B) EF (TMT-A and TMT-B) ME (RAVLT) VF (COWAT)	ATT (DST-F: F=2.921, *P*=.06; DST-B: F=5.766, *P*=.005) EF (TMT-A: F=0.837, *P*=.44; TMT-B: F=0.677, *P*=.51) ME (F=4.727, *P*=.01) VF (F=3.028, *P*=.06)
**CT+electroacupuncture**					
	Kim et al [42], 2020	16 (16)	CT only	GC (ADAS-Cog-K^bi^ and MoCA-K^bj^)	GC: (ADAS-Cog-K: Z=−0.38, *P*=.70; MoCA-K: Z=−0.72, *P*=.47)
**CT+PI**					
	Stuerz et al [68], 2022	26 (24)	Reverse sequential control	ATT (ACT) GC (MMSE)	ATT: ACT (η2<0.01, *P*=.21) GC: MMSE (η2=0.05, *P*=.09)
	Jesus et al [68], 2023a	27 (24)	IN	ATT (digit symbol coding of WAIS-III) GC (ACE-R^bk^) ME (ACE-R; WL^bl^ and LM of WMS-III)	ATT (F1,49=11.64; η2=0.19, *P*=.001) GC (F1,49=4.70; η2=0.09, *P*=.04) ME: ACE-R (F1,49=7.01; η2=0.13, *P*=.01); WL (F1,49=23.76; η2=0.33, *P*<.001); LM (F1,49=10.98; η2=0.18, *P*=.002)
	Jesus et al [69], 2023b	98 (101)	IN	ATT (digit symbol coding of WAIS-III) GC (ACE-R) ME (LM of WMS-III)	ATT (Cohen d=0.41, *P*<.001) GC (Cohen d=0.33, *P*<.001) ME (immediate recall: Cohen d=0.42, *P*<.001; delayed recall: Cohen d=0.35, *P*<.001)
**CT+current stimulation**					
	Gonzalez et al [60], 2021	22 (24; 21)	Sham tDCS^bm^+CT CT only	ATT (TMT-A) GC (MoCA) ME (DST and RBMT, 3rd edition)	ATT: TMT-A (F=0.64, *P*=.62) GC: MoCA (F=0.34, *P*=.85) ME: DST (F=1.99, *P*=.09); RBMT-3 (F=0.13, *P*=.96)
	Jones et al [62], 2023	13 (12)	PC+CT	ATT (ACE-X^bn^) ME (ACE-X)	ATT (Cohen d=0.94, *P*=.047) ME (η2=0.05, *P*=.35)
	Martin et al [53], 2019	33 (35)	CT+PC	ME (PALT^bo^ and RVIP^bp^) PS (SDMT) Subjective cognitive functioning (CFQ^bq^)	ME: PALT (F=0.26, *P*=.77); RVIP (F=0.2, *P*=.82) PS (F=1.39, *P*=.25) Subjective cognitive functioning (F=1.25, *P*=.29)
	Lau et al [44], 2024	11 (11)	CT+PC	GC (MoCA) EF (TMT-A, TMT-B, and Tower of London) ME (N-back task and CVVLT)	GC: MoCA (η2=0.05, *P*=.35) EF: TMT-A (η2=0.18, *P*=.06); TMT-B (η2=0.12, *P*=.13) Tower of London (η2=0.03, *P*=.42); ME: N-back (1-back: η2=0.17, *P*=.07; 2-back: η2=0.16, *P*=.07); CVVLT (verbal memory: η2=0.12, *P*=.07; delayed recall: η2=0.02, *P*=.59)
	Senczyszyn et al [47], 2023	13 (13; 12)	Current stimulation only Sham current stimulation only	GC (CANTAB^br^) VF (Verbal Fluency FAS test)	GC: CANTAB (Swms6: η2=0.042, *P*>.05; Palta4: η2=0.019, *P*=.03; Prmpci: η2=0.091, *P*=.02) VF (η2=0.042, *P*>.05)
**PT+current stimulation**					
	Liao et al [45], 2021	10 (10)	PT+PC	GC (MoCA) EF (TMT-A and TMT-B) ME (change detection task and CVVLT)	GC: F1,18=0.246; η2=0.246, *P*=.63 EF: TMT-A (F1,18=1.022; η2=0.054, *P*=.33); TMT-B (η2=0.271, *P*=.02) ME: change detection task (F1,18=1.046; η2=0.058, *P*=.32); CVVLT (F1,18=0.024; η2=0.001, *P*=.88)
	Xu et al [67], 2023	44 (49; 44; 43)	PT+sham current stimulation Sham PT+current stimulation; sham PT+sham current stimulation	ATT (TAP^bs^) GC (MoCA) EF (SCWT) ME (AVLT, ROCF, and Chinese Wechsler Memory Scale—Revised MQ^bt^)	ATT (auditory: F=2.66, *P*=.05; visual: F=4.536, *P*=.004; sustained reaction: F=3.609, *P*=.02) GC (F=7.415, *P*<.001) EF (F=.058, *P*=.98) ME: AVLT (immediate recall: F=3.207, *P*=.03; delayed recall: F=1.13, *P*=.34); ROCF (copy: F=2.489, *P*=.062; recall: F=0.571, *P*=.64); MQ (F=3.584, *P*=.02)

^a^PT: physical training.

^b^CT: cognitive training.

^c^IN: inactive control.

^d^GC: global cognition.

^e^COWAT: Controlled Oral Word Association Test.

^f^DSCT: Digit Symbol Substitution Test.

^g^HVLT: Hopkins Verbal Learning Test.

^h^TMT-A: Trail Making Test–A.

^i^TMT-B: Trail Making Test–B.

^j^SCWT: Stroop Color-Word Test.

^k^EF: executive function.

^l^DST: digit span test.

^m^ME: memory.

^n^WMS-LM I: Wechsler Memory Scale-Revised–Logical Memory I.

^o^WMS-LM II: Wechsler Memory Scale-Revised–Logical Memory II.

^p^RAVLT: Rey Auditory Verbal Learning Test.

^q^PS: processing speed.

^r^PC: placebo control.

^s^ATT: attention.

^t^SDMT: Symbol Digit Modalities Test.

^u^GC: global cognition.

^v^ADAS-Cog: Alzheimer Disease Assessment Scale–Cognitive.

^w^WAIS-III: Wechsler Adult Intelligence Scale–III.

^x^BVRT: Benton Visual Retention Test.

^y^LM: logical memory.

^z^WMS-III: Wechsler Memory Scale–III.

^aa^VF: verbal fluency.

^ab^AVLT: Auditory Verbal Learning Test.

^ac^DSST: Digit Symbol Substitution Test.

^ad^VFT: Verbal Fluency Test.

^ae^Italicized values indicate statistical significance.

^af^BD: block design.

^ag^WAIS-IV: Wechsler Adult Intelligence Scale–IV.

^ah^DST-F: Digit Span Test–Forward.

^ai^DST-B: Digit Span Test–Backward.

^aj^DSS: digit span sequence.

^ak^WLL: Word-List Learning Test.

^al^VFT-Letter: Verbal Fluency Test–Letter.

^am^VFT-Category: Verbal Fluency Test–Category.

^an^ACE: Addenbrooke Cognitive Examination.

^ao^MMSE: Mini-Mental State Examination.

^ap^DRT-II: Disjunctive Reaction Time.

^aq^HE: health education.

^ar^KMMSE: Korean version of the Mini-Mental State Examination.

^as^EFPT-K: Executive Function Performance Test.

^at^FAB: Frontal Assessment Battery.

^au^CDR-SOB: Clinical Dementia Rating Sum of Boxes.

^av^CMMSE: Mini-Mental State Examination, Chinese version.

^aw^STT-A: Shape Trail Test–A.

^ax^STT-B: Shape Trail Test–B.

^ay^CFT: Complex Figure Test.

^az^BNT-30: Boston Naming Test.

^ba^MoCA: Montreal Cognitive Assessment.

^bb^EXIT-25: Executive Interview-25.

^bc^CVVLT: Chinese version of the Verbal Learning Test.

^bd^HK-MoCA: Montreal Cognitive Assessment, Hong Kong version.

^be^NI: nutritional intervention.

^bf^PI: psychosocial intervention.

^bg^TEA: Test of Everyday Attention.

^bh^RBMT: Rivermead Behavioral Memory Test.

^bi^ADAS-Cog-K: Alzheimer Disease Assessment Scale–Cognitive, Korean version.

^bj^MoCA-K: Montreal Cognitive Assessment, Korean version.

^bk^ACE-R: Addenbrooke Cognitive Examination–Revised.

^bl^WL: waitlist control.

^bm^tDCS: transcranial direct current stimulation.

^bn^ACE-X: Adaptive Cognitive Evaluation-Explorer

^bo^PALT: Paired Associative Learning Test.

^bp^RVIP: Rapid Visual Information Processing.

^bq^CFQ: Cognitive Failures Questionnaire.

^br^CANTAB: Cambridge Neuropsychological Test Automated Battery.

^bs^TAP: Test Of Attentional Performance.

^bt^MQ: memory quotient.

#### The Combination of PT and CT

Among these 18 studies, 4 used a single-modal active control group [34,59,63,70], 3 compared against a health education group [15,40,71], and 5 used a passive control group [51,52,57,72,73]. Two studies used both a single-modal active control and a passive control group [65,66]. One study compared results with 2 single-modal active controls and a placebo group [38], while another used 3 single-modal active control groups [74]. In addition, 1 study contrasted results with 2 single-modal active controls, a health education group, and a passive control group [16]. Another study used a bimodal active control group [36].

Among these studies, 7 reported significantly greater improvements in all measured cognitive functions within the bimodal intervention groups [15,35,40,52,59,63,72]. Three studies revealed improvements in both bimodal intervention and control groups [16,37,61,70]. One study found greater improvements in executive function, global cognition, and verbal fluency within the PT-only control group, whereas attention and processing speed improved significantly across all groups [38]. Another study reported no changes in cognitive function across any group [57]. In addition, 1 study observed improvements in attention, episodic memory, and working memory across all groups but no changes in global cognition [74]. Finally, 1 study reported greater improvement in global cognition within the NI-only control group [65].

#### Three Studies Investigated the Combination of PT and PI

Among these 3 studies, 2 studies compared results with a single-modal active control group [39,48], and one compared with a passive control group [41]. One study found that both the bimodal intervention and control groups enhanced attention, memory, and processing speed, but only the intervention group significantly improved executive function [39]. Another study revealed improvements in global cognition only within the bimodal intervention group, although both groups improved in executive function [41]. In addition, one study reported significantly greater improvements in global cognition exclusively in the bimodal intervention group [48].

#### Two Studies Investigated the Combination of PT and NI

One study compared the intervention group with 2 single-modal active control groups and a health education control group [56]. It found that both the bimodal intervention and PT-only groups showed greater improvements in executive function and global cognition, although no gains were observed in memory or verbal fluency across groups [56]. Conversely, another study contrasted the intervention group with 2 single-modal active control groups [58] and revealed improvements in global cognition in all groups [58].

#### One Study Investigated the Combination of CT and Electroacupuncture

This study compared the intervention group with a single-modal active control group. It found that all groups demonstrated significant improvements in global cognition [42].

#### Three Studies Investigated the Combination of CT and PI

One study compared a reverse sequential control group [68], whereas 2 studies compared results with a passive control group [61,69]. Results from the latter 2 studies revealed significantly greater improvements in attention, episodic memory, and global cognition in the bimodal intervention group [61,69]. Conversely, the study with a reverse sequential control group found enhanced global cognition in both groups. Notably, the group starting with the PI showed more significant attention improvements after the initial training, while the group beginning with CT exhibited greater improvements following the second, combined training phase [68].

#### Five Studies Investigated the Combination of CT and Current Stimulation

Two of these studies compared the intervention group with a bimodal active control group [53,62]. One study contrasted the intervention group with a single-modal and a bimodal active control group [60]. Another study used a combination of CT and sham current stimulation as a control group [44]. One study included a current stimulation–only group, a sham current stimulation–only group, and a sham current stimulation control group [47].

One study observed improvements in everyday memory and global cognition across all groups, with attention improvements unique to the bimodal intervention group [60]. However, the CT-only control group showed greater working memory improvements than the bimodal intervention group [60]. Notably, transcranial direct current stimulation (tDCS) appeared to enhance the efficacy of CT in processing speed [60], despite not being a direct target. Another study showed that the bimodal intervention group demonstrated more significant attention improvements than both the placebo and CT groups [62]. In addition, 1 study found improvements in attention; processing speed; subjective cognitive functioning; and verbal, visual, and working memory across all groups [53]. Another study revealed improvements in episodic memory, executive function, and visual memory in both bimodal and active control groups [44].

#### Two Studies Investigated the Combination of PT and Transcranial Brain Stimulation

One study compared the intervention group with a bimodal active control group (PT and sham brain stimulation) [45], while another study used 2 bimodal active controls (PT and sham stimulation and sham PT with brain stimulation) along with a passive control group [67].

Results showed improvements in episodic memory, executive function, global cognition, and visual working memory in both the bimodal intervention and control groups [45]. However, the bimodal intervention group displayed greater enhancements across all cognitive functions compared to the control groups [67].

### Trimodal Interventions

#### Overview

Studies comparing combined PT, CT, and SA with health education controls reported significantly better cognitive improvements, especially in the memory domain [55,75,76]. Similarly, 3 out of 5 studies using combined PT, CT, and NI [46,49,54] showed significantly greater improvements in global cognition compared to control groups (Table 3).

**Table 3:** Studies that incorporated trimodal intervention and outcomes (k=8).

Study		Sample size (control sample size)	Control	Measured cognitive domains	Clinical outcomes
**PT^a^+CT^b^+NI^c^**					
	Bray et al [50], 2023	18 (15; 20; 15; 14)	PT+CT+PC^d^ PT+NI+PC PT+PC+PC PC only	GC^e^ (ADAS-Cog^f^) EF^g^ and ME^h^ (ADAS-Cog, TMT-A^i^, and TMT-B^j^)	The intervention group did not show significant differences compared to other groups across all tests (*P*>.05)
	Kobe et al [43], 2016	13 (9)	NI^k^	GC (AVLT^l^, DST^m^, VFT^n^, TMT-A, TMT-B, and SCWT^o^)	No significant difference between the two groups for all tests (*P*>.05)
	Montero-Odasso et al [54], 2023	34 (35; 37; 35)	PT+CT+sham NI PT+sham CT+NI PT+sham CT+sham NI Sham PT+sham CT+sham NI	GC (ADAS-Cog) EF (ADAS-Cog Plus variant)	GC (mean difference −2.64, *P*=.005^p^, Cohen d=0.71) EF (ADAS-Cog Plus variant: no significant improvement; *P*>.05)
	Phoemsapthawee et al [46], 2022	20 (20; 18)	PT only PC	GC (MMSE^q^) EF (TMT-A and TMT-B) ME (DST-F^r^ and DST-B^s^)	GC (F=13.158; η2=0.328, *P*<.001) EF: TMT-A (F=30.142; η2=0.527, *P*<.001); TMT-B (η2=0.376, *P*<.001) ME: DST-F (F=17.208; η2=0.389, *P*<.001); DST-B (η2=.495, *P*<.001)
	Yang et al [49], 2022	55 (57)	IN	GC (MoCA^t^)	GC: group×time interaction (χ²3=303.928; *P*<.001)
**PT+CT+SA**					
	Bae et al [55], 2019	41 (42)	HE^u^	GC (MMSE) EF (TMT-A and TMT-B) ME (spatial span task)	GC (*P*=.14) EF: TMT-A (*P*=.43); TMT-B (*P*=.68) ME (*P*=.02)
	Lee et al [75], 2023	140 (140)	HE	ATT^v^ and EF (TMT-A and TMT-B) ME (NCGG-FAT^w^) PS^x^ (DSST^y^)	ATT and EF: TMT-A (F=0.25); TMT-B (F=0.00) ME (F=5.04, *P*<.05) PS (F=0.67)
	Lin et al [76], 2020	61 (61)	HE	ATT (SLUMS^z^) GC (SLUMS) EF (SLUMS)	ATT (Wald χ2=50.84, *P*<.001) GC (Wald χ2=252.81, *P*<.001) EF (Wald χ2=115.99, *P*<.001)

^a^PT: physical training.

^b^CT: cognitive training.

^c^IN: inactive control.

^d^PC: placebo control.

^e^GC: global cognition.

^f^ADAS-Cog: Alzheimer Disease Assessment Scale–Cognitive.

^g^EF: executive function.

^h^ME: memory.

^i^TMT-A: Trail Making Test–A.

^j^TMT-B: Trail Making Test–B.

^k^NI: nutritional intervention.

^l^AVLT: Auditory Verbal Learning Test.

^m^DST: Digit Span Test.

^n^VFT: Verbal Fluency Test.

^o^SCWT: Stroop Color-Word Test.

^p^Italicized values indicate statistical significance.

^q^MMSE: Mini-Mental State Examination.

^r^DST-F: Digit Span Test–Forward.

^s^DST-B: Digit Span Test–Backward.

^t^MoCA: Montreal Cognitive Assessment.

^u^HE: health education.

^v^ATT: attention.

^w^NCGG-FAT: National Center for Geriatrics and Gerontology–Functional Assessment Tool.

^x^PS: processing speed.

^y^DSST: Digit Symbol Substitution Test.

^z^SLUMS: Saint Louis University Mental Status Examination.

#### Five Studies Investigated the Combination of PT, CT, and NI

One study compared the trimodal intervention group with an NI-only active control group [43], another compared it with both a PT-only active control and a placebo group [46], and a third study used an inactive control group [49]. One study contrasted outcomes with 4 control groups: combined PT and CT with placebo NI; combined PT and NI with sham CT; combined PT with sham CT and sham NI; and an inactive control group [54]. The fifth study compared the trimodal intervention with various combinations of PT, CT, and NI placebos [50].

In the first study, the trimodal intervention group study reported no substantial changes in cognitive functions across all groups [43]. Conversely, the second study found the intervention group showed significantly greater improvements in all cognitive functions compared with the placebo group, but not when compared to the PT-only control group [46] and the third study observed more significant enhancements in global cognition in the trimodal intervention group [49]. The fourth study also found greater improvements in global cognition in the trimodal intervention group, but no significant changes in other measures [54]. In the last study, the trimodal intervention group showed no significant difference compared to the controls [50].

#### Three Studies Investigated the Combination of PT, CT, and SA

All 3 studies contrasted the intervention group with a health education control group, revealing significant improvements in global cognition and memory for the trimodal intervention group [55,76]. In addition, 1 study found significant enhancements in memory compared to control groups [55], while another reported significant improvements in both attention and executive function during a 12-month follow-up in the intervention group [76].

### Quadrimodal Interventions

Most studies incorporating quadrimodal interventions reported small to moderate effects across various cognition domains, especially in global cognition (Table 4).

**Table 4:** Studies that incorporated quadrimodal interventions and outcomes (n=3).

Study		Sample size (control sample size)	Control	Measured cognitive domains	Clinical outcomes
**PT^a^+CT^b^+PI^c^+SA^d^**					
	Maffei et al [77], 2017	55 (58)	IN^e^	GC^f^ (ADAS-Cog^g^)	GC (Effect Size=−0.55, *P*<.001^h^)
	Straubmeier et al [64], 2017	208 (154)	IN	GC (MMSE^i^)	GC (Cohen d=0.26, *P*=.01)
**PT+CT+PI+NI^j^**					
	Liu et al [78], 2023	86 (106)	IN	GC (MMSE and MoCA^k^) ME (AVLT^l^ and PALT^m^)	GC (Hedges g=0.40, *P*=.03) ME (*P*=.05)

^a^PT: physical training.

^b^CT: cognitive training.

^c^PI: psychosocial intervention.

^d^SA: social activities.

^e^IN: inactive control.

^f^GC: global cognition.

^g^ADAS-Cog: Alzheimer Disease Assessment Scale–Cognitive.

^h^Italicized values indicate statistical significance.

^i^MMSE: Mini-Mental State Examination.

^j^NI: nutritional intervention.

^k^MoCA: Montreal Cognitive Assessment.

^l^AVLT: Auditory Verbal Learning Test.

^m^PALT: Paired Associative Learning Test.

#### Two Studies Investigated the Combination of PT, CT, PI, and SA

The 2 studies compared a quadrimodal intervention group with a passive control group [64,77]. One study found significantly greater improvements in global cognition in the quadrimodal intervention group [77]. In contrast, the other study reported no significant improvement in cognitive function within the intervention group, but the control group experienced deterioration [64].

#### One Study Investigated the Combination of PT, CT, PI, and NI

Compared to a passive control group, the quadrimodal intervention group showed significantly larger posttreatment improvements in global cognition and memory. However, these effects showed no difference between the two groups at the 12-month follow-up [78].

### The Use of Technology in MCI Interventions

Between 2015 and 2024, a total of 16 studies incorporated technology to enhance intervention delivery. The Nintendo Wii (n=1) was first used as a hardware platform for delivering PT [16]. Subsequent studies used various technologies, including virtual reality (VR) technology (n=1) for combined PT and CT intervention [37]; Nintendo Switch (n=1) [44]; and iPad (n=2) [50,57] for sensorimotor, visuomotor [54], and CT [50], respectively.

In addition, 13 studies used a range of computerized programs for CT targeting functions such as attention, memory, and executive function. These included AKL-T01 (n=1) [62], Brain Fitness (n=1) [16], COGPACK (n=2) [53,68], CogniPlus (n=1) [32], NeuronUP (n=1) [60], Neuropeak (n=1) [54], and RehaCom (n=3) [42,47,63] and 2 studies with no specified programs [44,49]. In addition, 1 study used the FitForAll program [16] for PT. Studies that incorporated CCT alongside other modalities showed significantly better improvements, particularly in global cognition, than studies with either active and inactive control groups. Details on tasks, duration, and major findings of these programs are presented in Multimedia Appendix 3.

Five studies incorporated tDCS with CT [44,47,53,60,62], while 2 studies combined tDCS with PT [45,67]. Specifically, they applied 1.5mA to 2 mA of tDCS to the left dorsolateral prefrontal cortex (DLPFC) or 1.5 mA of transcranial alternating current stimulation (tACS) on the prefrontal cortex [62].

### Acceptability and User Experience

User compliance is influenced by the acceptability and experience of treatments. Four studies indicated high acceptability and positive user experiences regarding multimodal interventions. Jesus et al [61] reported that 24 out of 27 participants demonstrated a 97% acceptability rate for the treatment effect and frequency of a combined CT and PI. Another study on a combined PT, CT, and PI intervention reported that 78% (39/50) of participants rated the program as “very good,” 20% (10/50) rated it as “good,” and 2% (1/50) rated it as “pleasurable” [68]. In addition, a study on a combined PT, CT, and SA program showed that 86% (35/41) of participants were satisfied with the intervention duration and 81% (33/41 participants) expressed a desire to continue [55].

Similarly, a study on a combined PT and CT intervention reported that 39 out of 44 (89%) participants perceived subjective benefits. In terms of overall satisfaction, 14 out of 44 (32%) participants rated it as “very good,” 24 out of 44 (54%) participants rated it as “good,” and 6 out of 44 (13%) participants rated it as “neither good nor bad.” In addition,12 out of 44 (27%) participants considered the user-friendliness of the intervention as “very good” [57].

### Dosage Effect and Health Economics

While treatment dosage may affect clinical outcomes, only 1 study evaluated the dosage effects of a quadrimodal intervention (combined PT, CT, PI, and SA) [64]. It found that 1 to 2 sessions per week of the quadrimodal intervention in daycare centers yielded no significant difference in cognitive outcomes compared with 3 to 5 sessions per week [64]. As such, the study suggested that less-frequent sessions (1-2 wk) might be as effective as more frequent sessions, thus further studies are warranted to validate these findings and test with other multimodal interventions.

In addition, while the cost-effectiveness of multimodal cognitive interventions is important for clinical practice, none of the included studies examined the cost-effectiveness of the identified intervention.

## Discussion

### Overview

This scoping review examines the current research landscape on nonpharmacological multimodal interventions for MCI, highlighting publication trends, intervention types, technology use, user experience, and dosage effects. Over the past decade, the variety of multimodal intervention combinations has increased. The most common interventions combine PT and CT, with additional components, such as NI, electroacupuncture, PI, and current stimulation. Cognitive outcome measures are diverse, targeting various domains. Bimodal and trimodal interventions generally outperform single-modal ones in improving global cognition, attention, and executive function. Notably, PT with current stimulation and PT with NI often demonstrate better cognitive improvements compared to active controls, while CT with PI shows more improvements compared to inactive controls. However, most studies on combined PT and CT report mixed results. Quadrimodal interventions also show superior improvements, although their long-term effect remains uncertain. This review, following the Arksey and O’Malley framework, did not conduct a meta-analysis or risk of bias assessments. The included studies suggested that trimodal and quadrimodal interventions, especially those including SA, might offer better cognitive outcomes, but findings should be interpreted cautiously due to the limited number of studies. Technologies, such as VR, gaming, computerized programs, and transcranial stimulation, are increasingly adopted in MCI interventions. Although technology-assisted training showed significant improvements in various cognitive domains comparable to traditional trainings, results should be interpreted with care given the limited studies. Future research should investigate whether technology-assisted training, with or without additional modalities, offers significantly better cognitive improvements than traditional training. Although only 4 included studies assessed the acceptability of multimodal interventions, they were generally well-received by users. Only 1 included study evaluated the dosage effects of a quadrimodal intervention, suggesting that 3 or more sessions per week may not be beneficial, although this finding should be interpreted cautiously. As none of the included studies evaluated the cost-effectiveness of these interventions, future studies should address this gap.

### Types of Modalities

Aside from traditional PT like aerobic and stretching exercises, recent research has been expanding to explore various PT types beyond traditional methods, including mind-body exercises such as tai chi [66], yoga [58], and dancing [15] as viable components in multimodal interventions for managing MCI.

Mind-body exercises, which engage the mind to influence bodily functions, are popular among older adults, partly due to cultural preferences in certain populations. The effectiveness of such exercises, such as tai chi, varies between Asian [74,79] and Western populations [39], highlighting cultural influences. tai chi, for instance, demands whole-body coordination, rhythmic movements, dynamic weight shifting, single-limb support, integrating movement recall (memorization and concentration), spatial orientation, and cognitive activities such as attention and executive control. This provides simultaneous PT and CT that could be beneficial for older adults with MCI [20,66]. In addition, these mind-body exercises offer relaxation and social support in group settings, potentially improving mood and motivation to participate in activities. Given their multifaceted benefits, mind-body physical activities may be incorporated into multimodal interventions for MCI.

With the growing interest in multimodal interventions, there is a noticeable increase in incorporating various interventions in addition to combined PT and CT. Modifiable lifestyle factors, including NI, are crucial for cognitive improvements among older adults with MCI. However, the effectiveness of NI as a standalone intervention has yielded mixed results for cognitive outcomes. Different dietary patterns and supplements have been suggested to promote cognitive health. For instance, the Mediterranean-Dietary Approaches to Stop Hypertension Intervention for Neurodegenerative Delay diet is popular. However, a large-scale study found that the Mediterranean-Dietary Approaches to Stop Hypertension Intervention for Neurodegenerative Delay diet was not significantly superior to a control diet with mild caloric restriction over 3 years [80]. In addition, systematic reviews suggest that certain single-nutrient supplements, such as folate, vitamin E, omega-3 fatty acids, and probiotics, show promising but preliminary results, often based on weak evidence or low-quality studies [81-83].

NI has drawn attention to its potential in multimodal interventions for MCI. All the included studies that incorporated NI demonstrated promising outcomes when combined with PT and CT compared to the control groups. However, some studies lacked detailed descriptions of their use of dietary guidelines, limiting the understanding of whether the specific dietary changes could be contributed to cognitive improvement. The current findings suggest that while a single NI may offer limited results, a combination of different modalities can provide a synergistic protective effect, highlighting the effectiveness of a multimodal approach over a single intervention.

In addition, there is an increasing use of PI and SA as supplementary components. Previous studies suggested that engaging in SA could help delay the development of dementia and cognitive decline [84,85]. SA and leisure activities not only have therapeutic benefits but also allow participants to engage with peers, fostering long-term adherence to interventions. It is important to note that the number of studies that incorporated the use of PI and SA is still relatively small; more studies are warranted to strengthen whether the implementation of additional PI and SA might provide additional benefits in a multimodal intervention for MCI.

Collectively, all the aforementioned intervention components play a role in enhancing mental health and cognitive functions across various aspects of MCI, suggesting that a larger number of combined modalities could be beneficial. This aligns with a previous systematic review that suggested an increased number of modalities might yield greater improvement in cognition [21]. Indeed, most of the included studies incorporating 3 or 4 modalities reported notable improvements in various cognitive domains, especially when additional modalities (including SA, NI, and PI) were combined with PT and CT. However, there is no consensus regarding the optimal number or combinations of modalities for multimodal interventions. Furthermore, it is worth noting that adopting multiple lifestyle changes may pose challenges for older adults. As Schneider and Yvon [86] suggested, such changes are more feasible for older adults in better health and with higher education levels. In current MCI management, while health advice is important, it may not sufficiently inform or motivate individuals with MCI to adopt new management strategies. Therefore, health education emerges as a crucial component for the successful implementation of lifestyle changes because it provides vital information on lifestyle modifications and guides the execution of physical or cognitive exercises at home.

Nonetheless, it is noteworthy that most of the included studies only compared the effectiveness of 3 or more modal combinations with single-modal interventions, health education, or passive control groups. It remains unclear whether there are more modalities or specific combinations that would offer greater benefit to individuals with MCI.

### The Current Use of Technology

Beyond traditional interventions, technology is increasingly embraced by researchers for its potential as a valuable asset in delivering multimodal interventions for clinicians and researchers from 2015 (n=1) [16] to 2023 (n=4) [47,54,62,63]. CCT comprises guided drill-and-practice on standardized tasks aimed at various cognitive domains that gradually progress with adjustable difficulty based on user performance. This approach is both cost-effective and safe for widespread use [87].

This review identified several computer programs that provide personalized CCT targeting various cognitive domains. Our findings concur with a previous systematic review, showing CCT and VR-CT as effective in enhancing attention, executive function, global cognition, memory, processing speed, verbal fluency, and visuospatial ability in older adults at risk of cognitive decline [25]. These outcomes are on par with traditional in-person PT and CT, suggesting CCT and VR-CT as promising ways for broad-scale cognitive intervention delivery.

Interactive video game–like programs offer training across multiple cognitive domains, including attention, executive function, processing speed, and visuomotor and visuospatial abilities, providing more engaging and motivating experiences for older adults with MCI than traditional face-to-face CT [26,88-90]. These programs also facilitate PT through diverse hardware. For instance, one included study used Nintendo Wii, Wii Remote, and Wii Balance Board for physical exercises (eg, aerobic exercises) [16]. Advanced technologies such as the latest Nintendo Switch, which tailored physical exercise games and hardware (eg, Ring Fit Adventure), adapt challenges to individual capacities, progressively improving cognitive function in older adults with MCI.

In addition to the computerized training program, the included studies that incorporated VR technology in delivering combined PT and CT demonstrated significant improvement in executive function and global cognition. These improvements were greater and consistent with the improvements from the combined PT and CT control group without the use of VR [36,37]. This positions VR as a promising tool for integrated therapy. As suggested by recent systematic reviews, VR could act as an assistive device to deliver training and help improve executive function and global cognition in individuals with MCI [25,88]. Compared to traditional PT and CT, VR-PT and VR-CT offer accessibility, cost-effectiveness, and immersive personalized experience to facilitate skill transfer to activities of daily living [36,37,90]. With advancements in technology, VR equipment has become more user-friendly, affordable, and suitable for home use and rehabilitation, benefiting older adults with mobility issues. However, potential side effects such as stimulator sickness, discomfort, tiredness, mood change induced by immersive experience, and some health-related issues from wearing the VR headgear [90] highlight the need for clinician oversight to adjust treatment as necessary.

Although the development of computerized training programs can be costly, the potential for widespread deployment can offset the expenses of hiring numerous qualified assessors or clinicians and the costs associated with traveling for face-to-face training. Future research should explore integrating these programs with multimodal interventions, such as NI, to enhance their effectiveness and improve the overall well-being of individuals with MCI.

MCI affects multiple cognitive functions. The DLPFC plays a crucial role in functional connectivity with other brain regions and controls various cognitive functions, including attention, decision-making, planning, and working memory. Research has found that individuals with MCI exhibit functional disconnection in the left DLPFC, leading to attention and working memory deficits [60]. This review included studies that used noninvasive brain stimulation techniques (eg, tDCS [45,53,60] targeting the left DLPFC and tACS targeting the prefrontal cortex); results from these studies suggested that using tDCS or tACS alongside PT or CT could enhance the effectiveness of PT or CT by improving cognitive capacity and reducing the load needed to perform cognitive tasks, thereby increasing processing speed and potentially serving as a viable component in future studies.

Taken together, technology stands as a valuable adjunct in administrating PT and CT, offering standardized, cost-effective, and assessable personalized experiences. Such technological integration could help researchers develop comprehensive management strategies for the widespread treatment of MCI in older adults.

### Acceptability and User Experience

Acceptability and user experience are pivotal for the success of interventions aimed at managing MCI, which directly influence its adherence rates. Adherence, in turn, significantly affects the effectiveness of multimodal interventions. Therefore, the interplay between user experience, acceptability, and adherence is vital in developing and implementing MCI interventions. However, most included studies have not investigated these factors, which may hinder clinicians or senior management in choosing or recommending treatment options. One included study suggested that the low adherence rate in older adults might be attributed to family responsibilities, health issues, and social commitments. Leveraging technology, particularly eHealth and mHealth tools, could improve adherence by alleviating barriers to adherence [65]. These tools could be used to facilitate easier implementation and monitoring of interventions, thereby reducing the time and cost required for broader deployment across various populations and regions [91]. Although none of the included studies have implemented such technological applications, future research should explore the potential of mobile technology in enhancing adherence in older adults with MCI.

### Limitations and Future Directions

While this scoping review offers a comprehensive overview of current study directions in multimodal interventions for MCI management, it has several limitations. First, by focusing solely on experimental studies with control groups, it may have overlooked relevant insights from qualitative research and gray literature. Second, this scoping review only included English articles, which may introduce cultural bias. Third, this review concentrated exclusively on the cognitive outcomes, omitting physical and psychological outcomes that might be crucial considerations for policy or clinical decisions. Fourth, several included studies had relatively small sample sizes, ranging from 19 to 27 participants [16,43,45,62,65]. Therefore, results should be interpreted with caution. Fifth, adhering to the Arksey and O’Malley framework and PRISMA-ScR guidelines meant no risk of bias assessments or evidence syntheses were conducted. Future systematic reviews or network meta-analyses should aim to summarize evidence regarding the relative effectiveness of different combinations of various multimodal interventions for MCI management.

While the current American Academy of Neurology guidelines for MCI management only recommend PT or CT, recent studies highlight the potential benefits of NI, PI, and SI for MCI management. With the annual conversion rate of MCI to dementia ranging from 10% to 15% per year [7], further research should explore various multimodal intervention combinations, focusing on follow-up, treatment duration, and frequency to potentially reduce or delay conversion rates. Although some included studies used brain imaging, most relied on self-reported or assessment scores, which might not accurately reflect real-world improvements. Future research should incorporate brain imaging technologies to validate results more robustly and elucidate the mechanisms underlying clinical improvements in cognitive function.

Considering the limited research on the cost-effectiveness of multimodal interventions for older adults with MCI and the growing aging population, future studies should investigate the economic viability of these interventions. In addition, the unclear dosage effects of different multimodal intervention combinations warrant investigation. Only one included study revealed that attending 1 or 2 sessions per week of a quadrimodal intervention was not significantly different from 3 to 5 sessions per week, suggesting that fewer sessions of quadrimodal intervention (PT, CT, PI, and SA) might still significantly improve global cognition [64]. However, this finding from a single study is not conclusive, further research is needed to determine the optimal dosage and combination of modalities to optimize the effectiveness of multimodal interventions for MCI.

Previous studies highlight the potential of lifestyle modifications such as dietary patterns in managing cognitive decline in older adults [92-94]. However, these studies used diverse nutrition supplements or dietary patterns. Similarly, while social engagement could help manage MCI [5], there is a dearth of studies on its effectiveness in enhancing physical and emotional well-being in older adults with MCI.

Finally, the included studies used diverse diagnostic criteria and cognitive measurements with different cutoff scores, potentially resulting in heterogeneous participant cohorts that could affect the results. An international consortium should be formed to develop standardized outcome measures for consistent outcome comparisons.

### Conclusions

This scoping review provides a comprehensive update on the use of multimodal interventions for improving cognitive functions in older adults with MCI. It presents study directions, multimodal intervention types, respective findings in the included studies, the role of technology in these interventions, and potential research directions. The most common multimodal intervention combines PT and CT. However, various types of interventions such as NI, PI, SI, and brain stimulation have also been incorporated into multimodal interventions more recently. Given the capacity of these interventions to stimulate multiple cognitive domains, the effectiveness of various combinations of modalities should be explored. The research gaps highlighted in this review pave the path for future large-scale clinical trials to help develop more effective management strategies for cognitive decline in older adults with MCI.

## Appendix

Multimedia Appendix 1 Search keywords and terms used in the current scoping review and results from each database.

Multimedia Appendix 2 Detailed table of the included studies’ and participants’ demographic characteristics.

Multimedia Appendix 3 Table of computerized training programs used in the included studies.

Multimedia Appendix 4 PRISMA-ScR (Preferred Reporting Items for Systematic Reviews and Meta-Analyses extension for Scoping Review) Checklist.

## Abbreviations

CCT: computerized cognitive training
CT: cognitive training
DLPFC: dorsolateral prefrontal cortex
MCI: mild cognitive impairment
NI: nutrition intervention
PI: psychosocial intervention
PRISMA-ScR: Preferred Reporting Items for Systematic Reviews and Meta-Analyses Extension for Scoping Reviews
PT: physical training
SA: social activities
tACS: transcranial alternating current stimulation
tDCS: transcranial direct current stimulation
VR: virtual reality
