# Supplementary material for: Nonpharmacological Multimodal Interventions for Cognitive Functions in Older Adults With Mild Cognitive Impairment: Scoping Review
Source: JMIR Aging. 2025 May 12;8:e70291. doi: 10.2196/70291 (PMC12107202; doi:10.2196/70291)
Supplement: Multimedia Appendix 1 [file aging_v8i1e70291_app1.docx]

**Appendix Table 3**. Search keywords and terms used in the current scoping review and results from each database.

**Date: 04/17/2024**

**Database: PubMed (Medline)**

| Set # |  | Results |
| --- | --- | --- |
| 1 | ("cognitive dysfunction"[MeSH Terms] OR "cognitive disord*"[Title/Abstract] OR "isolated memory impair*"[Title/Abstract] OR "incipient dementia"[Title/Abstract] OR "dementia prodrome"[Title/Abstract] OR "cognitive disabilit*"[Title/Abstract] OR "cognitive deficits"[Title/Abstract]) OR (mild cognitive impairment[MeSH Terms]) | 68,986 |
| 2 | "aged"[MeSH Terms] OR "geriatrics"[MeSH Terms] OR "elder*"[Title/Abstract] OR "old"[Title/Abstract] OR "old age"[Title/Abstract] OR "centenarian*"[Title/Abstract] OR "aging"[MeSH Terms] OR "aged adult*"[Title/Abstract] OR "aged individual*"[Title/Abstract] OR "aged population"[Title/Abstract] OR "older population"[Title/Abstract] OR "older person"[Title/Abstract] OR "senior*"[Title/Abstract] OR "older people"[Title/Abstract] OR "older adult*"[Title/Abstract] | 4,906,474 |
| 3 | "combin*"[Title/Abstract] OR "multi*"[Title/Abstract] OR "mix"[Title/Abstract] OR "integrat*"[Title/Abstract] OR "two"[Title/Abstract] OR "dual*"[Title/Abstract] OR "comprehensive*"[Title/Abstract] | 11,219,327 |
| 4 | "intervent*"[Title/Abstract] OR "train*"[Title/Abstract] OR "modal*"[Title/Abstract] OR "program*"[Title/Abstract] OR "therap*"[Title/Abstract] OR "treatment*"[Title/Abstract] OR "model*"[Title/Abstract] OR "rehabilitat*"[Title/Abstract] OR "task*"[Title/Abstract] OR "exercis*"[Title/Abstract] | 12,928,042 |
| 5 | #1 AND #2 AND #3 AND #4 | 8,670 |
| 6 | #5 ((randomized controlled trial[Filter])((Enligsh)[Filter]) | 704 |

**Date: 04/17/2024**

**Database: CINAHL Complete**

| Set # |  | Results |
| --- | --- | --- |
| 1 | (MH mild cognitive impairment OR AB cognitive dysfunction OR AB cognitive disord* OR AB isolated memory impair* OR AB incipient dementia OR AB dementia prodrome OR AB cognitive disabilit* OR AB cognitive deficit*) OR (MH mild cognitive impairment OR AB cognitive dysfunction OR AB cognitive disord* OR AB isolated memory impair* OR AB incipient dementia OR AB dementia prodrome OR AB cognitive disabilit* OR AB cognitive deficit*) | 19,639 |
| 2 | (TI older adult* OR TI older people OR TI elder* OR TI aged adult* OR TI  aged OR TI geriatric* OR TI aging OR TI aged adult* OR TI aged individual* OR TI older ag*) OR （AB older adult* OR AB older people OR AB elder* OR AB aged adult* OR AB aged OR AB geriatric* OR AB aging OR AB aged adult* OR AB aged individual* OR AB older ag* | 491,477 |
| 3 | (TI combin* OR TI multi* OR TI mix* OR TI integrat* OR TI two OR TI dual* OR TI comprehensiv*) OR (AB combin* OR AB multi* OR AB mix* OR AB integrat* OR AB two OR AB dual* OR AB comprehensive*) N5 (TI intervention* OR TI train* OR TI modal* OR TI program* OR TI therap* OR TI treatment* OR TI model* OR TI rehabilitat* OR TI task* OR TI exercise*) OR (AB intervention* OR AB train* OR AB modal* OR AB program* OR AB therap* OR AB treatment* OR AB model* OR AB rehabilitat* OR AB task* OR AB exercise*) | 2,448,704 |
| 4 | (TI randomized controlled trial* OR AB randomized controlled trial* ) | 123,272 |
| 5 | #1 AND #2 AND #3 AND #4 | 230 |

**Date: 04/17/2024**

**Database: Embase**

| Set # |  | Results |
| --- | --- | --- |
| 1 | ('mild cognitive impairment')/exp OR (('cognitive dysfunction'):ab,ti) OR (('cognitive disord*'):ab,ti) OR (('cognitive disabili*'):ab,ti) OR (('cognitive defici*'):ab,ti) OR (('cognitive defect*'):ab,ti) OR (('isolated memory impair*'):ab,ti) OR (('incipient dementia'):ab,ti) OR (('dementia prodrome'):ab,ti) | 115,487 |
| 2 | (aged)/exp OR (('older people'):ab,ti) OR (('aged adult*'):ab,ti) OR ((elder*):ab,ti) OR ((geriatric*):ab,ti) OR ((aging):ab,ti) OR (('aged individual*'):ab,ti) OR (('older ag*'):ab,ti) | 4,324,447 |
| 3 | (combin* OR multi* OR mix* OR integrat* OR two OR dual* OR comprehensive*) | 16,242,784 |
| 4 | (intervent* OR train* OR modal* OR program* OR therap* OR treatment* OR model* OR rehabilitat* OR task* OR exercis*) | 22,716,860 |
| 5 | ('randomized controlled trial')/exp OR (('randomized controlled trial'):ab,ti) OR ((randomized controlled trial)/br) | 1,178,952 |
| 6 | #1 AND #2 AND #3 AND #4 AND #5 | 1570 |

**Date: 04/17/2024**

**Database: Web of Science**

| Set # |  | Results |
| --- | --- | --- |
| 1 | TS=(mild cognitive impairment OR cognitive dysfunction OR cognitive disord* OR cognitive disabili* OR cognitive defici* OR cognitive defect* OR isolated memory impair* OR incipient dementia OR dementia prodrome) | 326,908 |
| 2 | TS=(aged OR older people OR aged adult*OR elder* OR geriatric* OR aging OR aged individual* OR older age*) | 4,563,975 |
| 3 | TS=(combin* OR multi* OR mix* OR integrat* OR two OR dual* OR comprehensiv* NEAR/5 intervent* OR train* OR modal* OR program* OR therap* OR treatment* OR model* OR rehabilitat* OR task* OR exercis* ) | 37,269,195 |
| 4 | (ALL=(randomized controlled trial*)) OR TS=(randomized controlled trial*) | 568,001 |
| 5 | #1 AND #2 AND #3 AND #4 | 6,795 |

**Date: 04/17/2024**

**Database: PsycINFO (via ProQuest)**

| Set # |  | Results |
| --- | --- | --- |
| 1 | tiab(Mild Cognitive Impairment) OR tiab(cognitive dysfunction) OR tiab(cognitive disord*) OR tiab(cognitive disabili*) OR tiab(cognitive defici*) OR tiab(cognitive defect*) OR tiab(isolated memory impair*) OR tiab(incipient dementia) OR tiab(dementia prodrome) OR tiab(mild cognitive) | 146,259 |
| 2 | tiab(aged) OR tiab(older people) OR tiab(aged adult*) OR tiab(elder*) OR tiab(geriatric*) OR tiab(aging) OR tiab(aged individual*) OR tiab(older adult*) OR tiab(older ag*) | 483,159 |
| 3 | tiab(combin*) OR tiab(multimodal) OR tiab(multi*) OR tiab(mix*) OR tiab(integrat*) OR tiab(two) OR tiab(dual*) OR tiab(comprehensiv*) | 1,688,151 |
| 4 | [tiab(intervention*) OR tiab(train*) OR tiab(modal*) OR tiab(program*) OR tiab(therap*) OR tiab(treatment*) OR tiab(model*) OR tiab(rehabilitat*) OR tiab(task*) OR tiab(exercis*)](https://www.proquest.com/recentsearches.recentsearchtabview.recentsearchesgridview.scrolledrecentsearchlist.checkdbssearchlink:rerunsearch/28C204E19CBE4060PQ/None?site=psycinfo&t:ac=RecentSearches) | 2,500,760 |
| 5 | [tiab(randomized controlled trial)](https://www.proquest.com/recentsearches.recentsearchtabview.recentsearchesgridview.scrolledrecentsearchlist.checkdbssearchlink:rerunsearch/A408117D4E8948FDPQ/None?site=psycinfo&t:ac=RecentSearches) | 55,545 |
| 6 | #1 AND #2 AND #3 AND #4 AND #5 | 591 |
