# Supplementary material for: Nonpharmacological Multimodal Interventions for Cognitive Functions in Older Adults With Mild Cognitive Impairment: Scoping Review
Source: JMIR Aging. 2025 May 12;8:e70291. doi: 10.2196/70291 (PMC12107202; doi:10.2196/70291)
Supplement: Multimedia Appendix 2 [file aging_v8i1e70291_app2.docx]

| **Appendix Table 2.** Demographic and Details of the Multimodal Studies Included (k = 49) | | | | | | | | | | | | |
| --- | --- | --- | --- | --- | --- | --- | --- | --- | --- | --- | --- | --- |
| Source(year) | Country | Place of recruitment | Subtype of MCI (Diagnostic Criteria) | Sample Size | Baseline cognition mean (SD) | Mean Age (SD) | Gender: Male (Female) | Types of intervention | | Duration | Total Duration | Timepoint on assessment |
| Bae et al. (2019) | Japan | Community | / | Multimodal: 41 | MMSE: 27.10 (2.10) | 71.6  (5.0) | 23(18) | Multimodal Intervention (Kenkojiseichi): Physical activities such as walking, muscle strength training combined with cognitive activities (such as arts and crafts), and social activities (such as socializing physically with others). | | 2 /wk, 90 min | 24 wk | Baseline, 24-wk |
|  |  |  |  | HE: 42 | MMSE  26.70 (2.00) | 76.4  (5.1) | 20(22) | HE: Classes on oral care and nutrition provided by a professional lecturer. | |  |  |  |
| Blumenthal et al. (2019) | The United States | Clinic and Community | / | Multimodal: 40 | MoCA:  25.00  (2.50) | 64.9  (6.2) | 14(26) | Multimodal intervention: Aerobic exercise of moderate intensity (e.g., walking or stationary biking), DASH nutrition instruction to meet DASH guidelines. | | PT: 3/wk,  35min  Nutrition:  1/wk for first 3-mo, 1/2wk for other 3-mo | 6 mo | Baseline, 6-mo, 12-mo follow-up |
|  |  |  |  | PTc: 41 | MoCA:  24.80  (2.70) | 65.8  (7.3) | 13(29) | PTc: Aerobic exercise of moderate intensity (e.g., walking or stationary biking) | |  |  |  |
|  |  |  |  | Nutrition Control: 41 | MoCA:  24.40  (2.70) | 66  (7.1) | 15(26) | Nutrition Control: DASH diet instruction to meet DASH guidelines | |  |  |  |
|  |  |  |  | HE:38 | MoCA:  24.40  (2.30) | 64.7  (6.6) | 12(26) | HE: Health education through phone calls, cardiovascular diseases health-related topics | |  |  |  |
| Bray et al. (2023) | Canada | Community | Albert et al. | Multimodal: 19 | MoCA:  22.94  (2.96) | 73.7  (6.9) | 9(10) | Multimodal Intervention: Combined intervention with CT, PT and Nutrition intervention. CT was provided through an iPad consisting of two different visuo-motor tasks for working memory and attention for 30 min. PT included 60 min of resistance training and aerobic exercise. Nutrition intervention included the use of Vitamin D3 tablet, participants were to ingest one tablet of 10,000 IU of Vitamin D3 three times per week. | | 3/wk, 90 min | 20 wk | Baseline, 6-mo, 12-mo follow-up |
|  |  |  |  | PT + CT + Sham nutrition: 16 | MoCA:  23.13  (2.45) | 73.3  (7.4) | 11(5) | Combined intervention with PT (60 mins of resistance training and aerobic exercise), CT (30 mins of iPad-based visuomotor task), and placebo Vitamin pills with the same dosage (three times per week) | |  |  |  |
|  |  |  |  | PT + Nutrition + CTc: 21 | MoCA: 22.90  (3.05) | 75.9  (7.6) | 11(10) | Combined intervention with PT (60 min of resistance training and aerobic exercise), CTc (30 min of controlled cognitive training in which participants were to conduct a pre-defined touristic search of a foreign city or watch a video on National Geographic through iPad), and Vitamin D3 tablet with the same dosage (one tablet, three times per week) | |  |  |  |
|  |  |  |  | PT + Sham Nutrition + CTc: 17 | MoCA: 22.82  (1.81) | 72.1  (4.2) | 12(5) | Combined intervention with PT (60 min of resistance training and aerobic exercise), placebo Vitamin pills, and CTc (30 mins of controlled cognitive training) | |  |  |  |
|  |  |  |  | PTc + CTc + sham Nutrition: 17 | MoCA:  22.12  (2.91) | 74.0  (5.77) | 4(13) | Active Control: Combined intervention with PTc (60 min of controlled physical training including stretching, balance, and toning exercise), 30 min of Ctc, and placebo Vitamin pills. | |  |  |  |
| Callisaya et al. (2021) | Australia | Clinic and Community | / | Multimodal: 44 | MoCA:  26.40 (2.40) | 72.9  (7.2) | 17(27) | Multimodal Intervention (The StandingTall Program): Physical exercise (balance and strength), combined with dual tasking (cognitive task added during the balance exercise through auditory and visual cues), and monthly health information. | | 2 hr/wk (40 min in wk 1 and 2, gradually increase to 120 from wk 9 onward) | 6 mo | Baseline, 6-mo |
|  |  |  |  | Inactive Control: 49 | MoCA:  26.10 (2.50) | 72.8  (6.9) | 22(27) | Inactive Control: Monthly health information fact sheet would be delivered through post | |  |  |  |
| Chobe et al. (2022) | India | Community | / | Multimodal: 24 | / | 63.2  (6.2) | 13(11) | Multimodal Intervention: Combined intervention with 60 min of Integrated Yoga training, and Ayurveda Rasayana intervention. Participants were advised to take 5 mL of Brahmi ghrita (a ghee-based polyherbal Ayurvedic formulation) along with 50 mL of warm milk or water on an empty stomach once a day. | | 6/wk, 60 min | 8 wk | Baseline, 8-wk |
|  |  |  |  | PTc: 25 | / | 62.4  (6.1) | 12(13) | PTc: 60 min of Integrated Yoga Only | |  |  |  |
|  |  |  |  | Nutrition Control: 23 | / | 64.4  (7.2) | 10(13) | Nutrition Control: Ayurveda Rasayana intervention only | | 1/day |  |  |
| Fairchild et al. (2023) | The United States | Community | Albert et al. | Multimodal:  36 | / | 71.0 (8.8) | 35(1) | Multimodal Intervention: Combined PT (30 min of aerobic exercise and 20 min of full-body resistance exercises) and group-based CT | | 2 to 3 / wk, 50 min | 6 mo | Baseline, 6-mo |
|  |  |  |  | Sham exercise + CT: 36 | / | 73.7 (10.1) | 34(2) | Group-based CT (non-mnemonic pre-training focused on imagery, semantic elaboration, and relaxation training) | |  |  |  |
| Singh et al. (2014) | Australia | Community | Petersen et.al 1999 | Mutlimodal: 27 | ADAS-Cog:  8.02 (1.15) | 70.1 (6.7)^a^ | 68%^a^ Female | Multimodal Intervention: Combined CCT (delivered through the COGPACK program, a computer-based multidomain cognitive exercise for neurorehabilitation targeting memory, executive function, attention, and processing speed) and progressive resistance training (PRT). | | 2 to 3 / wk, 100 mins | 6 mo | Baseline, 6-mo, 18-mo follow-up |
|  |  |  |  | Sham exercise + CCT: 22 | ADAS-Cog:  8.79 (1.23) |  |  | Combined intervention with Sham exercise (stretching and seated calisthenics) and CCT. | | 2 to 3 / wk, 75 min |  |  |
|  |  |  |  | PRT + Sham CT: 24 | ADAS-Cog:  8.29 (1.27) |  |  | Combined intervention with PRT and sham CT (watching 5 short National Geographic videos and answering a set of 15 questions). | | 2 to 3 / wk, 75 min |  |  |
|  |  |  |  | Sham exercise + Sham CT: 27 | ADAS-Cog:  8.09 (1.14) |  |  | Sham exercise and sham CT | | 2 to 3 / wk, 60 min |  |  |
| Fogarty et al. (2016) | The United States | Clinic | sd & md-aMCI: Petersen et.al 2004 | Multimodal: 22 | MMSE: 28.33 (1.39)  MoCA:  23.71 (1.82) | 71.6  (9.3) | 12(10) | Multimodal Intervention: TTC and Memory Intervention Program (MIP) consisting of 8 sessions, 6 of which focus on education on lifestyle factors that impact memory and memory strategies. The remaining two sessions are follow-up sessions at month one and month three. | | 6 sessions of MIP with 2 follow up at month 1 and month 3  For TTC: 2/wk, 90 min | 10 wk | Baseline, 10-wk, 22wk follow-up |
|  |  |  |  | HE: 18 | MMSE:  27.88 (1.05)  MoCA:  24.83 (2.04) | 65.8  (7.3) | 9(9) | HE: MIP alone | |  |  |  |
| Gill et al. (2015) | Canada | Community | / | Multimodal: 21 | MMSE:  28.70 (1.00)  MoCA:  35.10 (2.10) | 72.6  (7.4) | 22(18) | Multimodal Intervention: PT (aerobic, strength, balance and flexibility training) for 60 to 75 min combined with dual tasking (answering cognitively challenging and randomly generated arithmetic questions) during square-stepping exercise for 45 min. | | 2 to 3 / wk | 26 wk | Baseline, 12-wk, 26wk, 52wk follow-up |
|  |  |  |  | PTc: 23 | MMSE:  28.90 (1.30)  MoCA:  24.70 (1.70) | 74.5  (7.0) | 19(21) | PTc: Physical training for 60 to 75 min with square-stepping exercise without dual tasking for 45 min. | |  |  |  |
| Gonzalez et al. (2021) | Hong Kong | Community | ECADWG | Multimodal: 21 | MoCA:  23.70 (1.70) | 69.8  (5.3) | 6(15) | Multimodal intervention: CCT (Neuron Up, an online platform that provides customizable cognitive training targeting executive function, working memory, and attention) for 30 min combined with tDCS (a constant current delivered at 1.5 mA along with a ramp-up for 30 sec and ramp-down for 30 sec on the left dorsolateral prefrontal cortex) during the cognitive training. | | 3/wk, 30 min | 3 wk | Baseline, 3-wk, 6-wk follow-up |
|  |  |  |  | Sham tDCS + CCT: 24 | MoCA:  24.10 (2.40) | 71.0  (6.2) | 8(16) | Sham tDCS (only consisting of the ramp-up and ramp-down procedures to mimic the physical sensation) was delivered during CCT. | |  |  |  |
|  |  |  |  | CCTc: 21 | MoCA:  24.30 (1.70) | 70.6  (5.4) | 4(17) | CCTc: CCT alone | |  |  |  |
| Griffiths et al. (2020) | Thailand | Community | Albert et al. | Multimodal: 35 | / | / | 11(24) | Multimodal Intervention: Physical Movement Activities (PMA), using body movement and two bamboo sticks to facilitate movement creating sound and rhythm combined with multifaceted cognitive training (MCT) that targets attention, memory, and executive function. | | 2/wk, 20 min for PMA in wk 1-4, 25 min in wk 5-8, 30 min in wk 9-12, and 60-90 min for MCT | 12 wk | Baseline, 12-wk |
|  |  |  |  | Inactive Control: 35 | / | / | 11(24) | Inactive Control: No Intervention | | / |  |  |
| Hagovská& Nagyova (2016) | Slovak Republic | Clinic | ICD-9-CM 331.83 | Multimodal: 40 | MMSE:  25.98 (2.57) | 68.2  (6.8) | 22(18) | Multimodal Intervention: Balance training (walking over obstacles, walking with a directional change, walking with a speed change, change of walking base, speed walking, walking with a load, and walking up and down the stairs) and CT using CogniPlus (subprogram: Alert, Nback, Names, Pland, and Vismo). | | CT: 2/wk, 30 min  PT: daily/wk, 30 min | 10 wk | Baseline, 10-wk |
|  |  |  |  | PTc: 40 | MMSE:  26.03 (1.47) | 66.0  (5.7) | 19(21) | PTc: Balance training only | | Daily/wk,30 min |  |  |
| Hagovská& Olekszyova (2016) | Slovak Republic | Clinic | ICD-9-CM 331.83 | Multimodal: 40 | MMSE:  25.97 (2.57) | 68.2  (6.7) | 22(18) | Multimodal Intervention: Balance training (walking over obstacles, walking with directional change and walking with a speed change, walking with a load and walking up and down the stairs) and CT using CogniPlus (subprogram: Alert, Nback, Names, Pland, and Vismo). | | CT: 2/wk, 30 min  PT: daily/wk, 30 min | 10 wk | Baseline, 10-wk |
|  |  |  |  | PTc: 40 | MMSE:  26. 02 (1.47) | 65.7  (5.6) | 19(21) | PTc: Balance training only | | Daily/wk,30 min |  |  |
| Hagovská& Olekszyova (2016) | Slovak Republic | Clinic | ICD-9-CM 331.83 | Multimodal: 40 | MMSE:  25.97 (2.57) | 68  (4.4) | 22(18) | Multimodal Intervention: Balance training (walk over obstacles, walk with direction change and walk with speed change, walk with load, and walk up and down the stairs) and CT using CogniPlus (subprogram: Alert, Nback, Names, Pland, and Vismo). | | CT: 2 per wk, 30 min  PT: daily/wk, 30 min | 10 wk | Baseline, 10-wk |
|  |  |  |  | PTc: 40 | MMSE:  26.02 (1.47) | 65.9  (6.2) | 19(21) | PTc: Balance training only | | Daily/wk,30 min |  |  |
| Hagovskáet al. (2016) | Slovak Republic | Clinic | ICD-9-CM 331.83 | Multimodal: 40 | MMSE:  25.90 (7.30) | 68.0  (4.4) | 22(18) | Multimodal Intervention: Dynamic balance training (walking over obstacles, walking with direction, walking with speed change, walking with bags, walking upstairs and downstairs, change of walking base, walking with step length change-stride length change, tandem walk, speed walk) and CT using CogniPlus (subprogram: Alert, Nback, Names, Pland, and Vismo). | | CT: 2 per wk, 30 min  PT: daily/wk, 30 min | 10 wk | Baseline, 10-wk |
|  |  |  |  | PTc: 40 | MMSE:  26.80 (6.80) | 65.9  (6.2) | 19(21) | PTc: Balance training only | | Daily/wk,30 min |  |  |
| Jeong et al. (2021) | Korea | / | Petersen et.al  1999 | Multimodal: 13 | MMSE:  25.77 (2.31) | 70.2  (7.5) | 4(9) | Multimodal Intervention: Physical activities (such as aerobic exercise) combined with cognitive tasks (such as counting and calculation) while doing exercise. | | 2 per wk, 90 min | 12 wk | Baseline, 12-wk |
|  |  |  |  | HE: 13 | MMSE:  25.00 (2.58) | 71.8  (5.5) | 4(9) | No intervention, maintaining daily life and monthly educational class | | Once pre month |  |  |
| Jesus et al. (2023)a | Portugal | Community | / | Multimodal: 27 | ACE-R:  66.22 (10.61) | 77.4  (5.2) | 10(17) | Multimodal Intervention (HEPPI): A home-based multimodal intervention consisting of CT (targeting attention and episodic memory), psychotherapeutic intervention, and compensatory strategies training. | | Once per wk, 10 min for initial mood check, 20 min for CT, 45 min for psychotherapeutic intervention or compensatory strategies training and 15 min for summary. | 10 wk | Baseline, 10-wk |
|  |  |  |  | Inactive Control: 24 | ACE-R:  65.46 (8.43) | 75.9  (4.6) | 6(18) | No intervention | | / |  |  |
| Jesus et al. (2023)b | Portugal | Community | / | Multimodal: 98 | / | 79.1 (5.4) | 12(86) | Multimodal Intervention (HEPPI): A home-based multimodal intervention consisting of CT (targeting attention and episodic memory), psychotherapeutic intervention, and compensatory strategies training. | | Once per wk, 90 min | 10 wk | Baseline, 10-wk, after 3 mo |
|  |  |  |  | Inactive Control: 101 | / | 80.3 (4.7) | 22(79) | Treatment as Usual | |  |  |  |
| Jones et al. (2023) | The United States | / | md-aMCI | Multimodal: 14 | MoCA:  25.57 (3.48) | 70.4  (6.5) | 5(9) | Multimodal Intervention: CCT (AKL-T01, a proprietary system based on patented technology underlying the NeuroRacer paradigm that challenges cognitive control by requiring multitasking performance combined with tACS (6 Hz, 1.5mA) delivered using a self-applied humm tACS device for 15 min with an additional of 30 sec ramp-up and 30 sec ramp-down. | | 5 per wk in wk 1, Onceper wk afterward, 16 min for tACS and 20 min for CCT | 4 wk | Baseline, 1-wk, after 1 mo |
|  |  |  |  | CTc + Sham tACS: 13 | MoCA:  25.55 (1.86) | 69.5  (4.5) | 9(4) | CCT + Sham tACS (1Hz). | | 5 per wk in wk 1, Onceper wk afterward, 16 min for Sham tACS and 20 min for CCT |  |  |
| Kamegaya et al. (2014) | Japan | Community | aMCI: Petersen et.al  2004 | Multimodal: 26 | MMSE:  27.60 (2.00) | 73.6  (5.6) | 2(24) | Multimodal Intervention: Physical exercise (muscle stretching and strengthening exercise in a sitting position, and muscle strengthening exercise in a standing position) and Leisure activities. | | Once per wk, averaging 45 min | 12 wk | Baseline, 12-wk |
|  |  |  |  | Inactive Control: 26 | MMSE:  27.90 (1.60) | 76.2  (6.1) | 3(23) | No intervention | |  |  |  |
| Kim & Park (2023) | Korea | Community | / | Multimodal: 21 | FAB:  9.90 (1.13) | 74.3  (5.4) | 10(11) | Multimodal Intervention: Physical and Cognitive dual-task training, such as counting numbers, and calculation while conducting physical tasks such as aerobic exercises, and strength exercises. | | 2 per wk, 45 min | 8 wk | Baseline, 8-wk |
|  |  |  |  | CCTc: 21 | FAB:  10.10 (1.37) | 74.7(5.6) | 9(12) | CCTs delivered through the RehaCom software targetting executive functioning. | |  |  |  |
| Kim et al. (2020) | Korea | Clinic | Petersen et.al  1999&2004 | Multimodal: 16 | MoCA:  18.75 (2.54) | 69.9(5.9) | 2(14) | Multimodal Intervention: Electroacupuncture treatment (EA) performed on Baihui (GV20), Sishencong (EX-HN1), Fengchi (GB20), and Shenting (GV24) combined with CCT delivered through the RehaCom software | | 3 per wk, 30 min for EA and 30 for CCT | 8 wk | Baseline, 8-wk, 20-wk follow-up |
|  |  |  |  | CCTc: 16 | MoCA:  19.31 (2.92) | 74.3 (5.4) | 2(14) | CCTs delivered through the RehaCom software. | | 3 per wk, 30 min |  |  |
| Köbe et al. (2016) | Germany | Clinic | sd & md- aMCI: Mayo Criteria | Multimodal: 13 | MMSE:  28.50 (1.10) | 70(7.2) | 9(4) | Multimodal Intervention: PT (aerobic exercise) combined with CCT (AKTIVA program), and nutrition intervention delivered through supplemental capsules with 2200 mg long-chain omega-3 FA per day (4 capsules of 1320 mg eicosapentaenoic acid (EPA), 880 mg docosahexaenoic acid (DHA), and 15 mg of vitamin E for six months before or at a main meal). | | 2 per wk, 45 min for PT. 90 min for CT starting at wk 4 | 6 mo | Baseline, 6-mo |
|  |  |  |  | Nutrition + PTc: 9 | MMSE:  27.90 (1.70) | 70(5.2) | 5(4) | Dietary intervention combined with Sham physical training (stretching and toning) | | 2 per wk, 45 mins |  |  |
| Lam et al. (2015) | Hong Kong | Community | / | Multimodal: 132 | MMSE:  25.20 (2.20) | 76.3(6.6) | 28(104) | Multimodal Intervention: The Integrated Cognitive-Physical group participates in one type of CT (such as playing a board game) and two types of mind-body exercises from the reference list. | | 3 wk, 60 min | 12 mo | Baseline, 4-mo, 8-mo, 12-mo |
|  |  |  |  | Social Support: 131 | MMSE:  25.60 (2.40) | 75.4(6.1) | 29(102) | The social support control group participates in at least three one-hour social activities from the reference list. | | At least 3 hr per wk |  |  |
|  |  |  |  | CTc: 145 | MMSE:  25.70 (2.40) | 74.4(6.4) | 30(115) | The cognitive control group participates in at least three cognitive activities per week. | | At least 3 hr per wk |  |  |
|  |  |  |  | PTc: 147 | MMSE:  25.80 (2.30) | 75.5(6.7) | 34(113) | The physical control group participates in one stretching and toning exercise, one mind-body exercise, and one aerobic exercise in a week. | | 3 hr per wk |  |  |
| Lau et al. (2024) | Taiwan | Clinic | Petersen et al. 2018 | Multimodal: 11 | MMSE: 24.80 (1.40) | 72(17.3) | 4(7) | Multimodal Intervention: CCT (cognitive training game on Nintendo Switch targeting executive function, working memory, and attention) for 40 min combined with tDCS (a constant current delivered at 2 mA along with a ramp-up for 30 sec and ramp-down for 30 sec on the left dorsolateral prefrontal cortex) during the cognitive training for 20 min | | 3 per wk, 40 min | 5 wk | Baseline, 5wk |
|  |  |  |  | Sham tDCS + CCT: 10 | MMSE: 26.00 (2.70) | 69(4.9) | 3(7) | CCT + Sham tDCS (2 mA) for 30 sec | |  |  |  |
| Lee et al. (2023) | Japan | Clinic | / | Multimodal: 140 | / | 76.3(4.1) | 81(59) | Multimodal Intervention: COGNICISE, a community-based multicomponent program consisting of dual-task exercise (conducting cognitive tasks while performing moderate aerobic exercise), combined with social engagements. | | Once per wk for dual-task exercise, 2 per month for mentally stimulating social activities | 10 mo | Baseline, 10-mo |
|  |  |  |  | HE: 140 | / | 76.4(4.2) | 88(52) | HE: Participants in the control group received an educational lesson and no information regarding the intervention | | 3 sessions, 60 min |  |  |
| Li et al. (2022) | China | Clinic | / | Multimodal: 48 | MMSE:  28.00 (1.40) | 65.6(5.6) | 14(34) | Multimodal Intervention: MiXT, consisting of CT (targeting short-term memory, mental rotation, speed perception, and motion trace perception), and PT (Tai Chi) | | 3 to 4 per wk, 20 to 30 min for CT and 120 min for PT | Class A: 12 mo  Class B: 24 mo | Baseline, 6-mo, 12-mo, 18-mo, 24-mo |
|  |  |  |  | CTc: 51 | MMSE:  27.80 (2.10) | 65.5(7.2) | 11(40) | CTc: CT only | | 3 to 4 per wk, 20 to 30 min |  |  |
|  |  |  |  | Inactive Control: 53 | MMSE:  27.80 (1.60) | 66.6(7.1) | 24(29) | Inactive Control: No intervention. General health advice only | | / |  |  |
| Liao et al. (2019) | Taiwan | Community | / | Multimodal: 18 | MMSE:  27.20 (1.90) | 75.5(5.2) | 7(11) | Multimodal Intervention: By using Virtual Reality (VR) using the Kinect system to deliver PT (such as simplified 24-form Yang-style Tai Chi, resistance exercise, aerobic exercise, and functionally oriented tasks) combined with VR-based CT through Cognitive training VR game, include the Job Stimulator. | | 3 per wk, 60 min | 12 wk | Baseline, 12-wk |
|  |  |  |  | PTc + CTc: 16 | MMSE:  27.20 (1.60) | 73.1(6.8) | 4(12) | Ptc + CTc: Combined PT with functional tasks and cognitive tasks. | |  |  |  |
| Liao et al. (2020) | Taiwan | Community | / | Multimodal: 18 | MMSE:  27.20 (1.90) | 75.5(5.2) | 7(11) | Multimodal Intervention: By using Virtual Reality (VR) using the Kinect system to deliver PT (such as simplified 24-form Yang-style Tai Chi, resistance exercise, aerobic exercise, and functionally oriented tasks) combined with VR-based CT through Cognitive training VR game, including Job Stimulator. | | 3 per wk, PT: 40 min  CT: 20 min | 12 wk | Baseline, 12-wk |
|  |  |  |  | PTc+CTc: 16 | MMSE:  27.20 (1.60) | 73.1(6.8) | 4(12) | PTc + CTc: Combined PT with functional tasks and cognitive tasks. | | 3 per wk, 60 min |  |  |
| Liao et al. (2021) | Taiwan | Clinic | Petersen et.al  2004 | Multimodal: 10 | MoCA:  23.30 (4.63) | 72.6(4.1) | 2(8) | Multimodal Intervention: Anodal tDCS (2mA of direct current) combined with Tai Chi (24 forms of Yang Style Tai Chi) | | 3 per wk, Anodal tDCS: 20 min  TC: 40 min | 12 wk | Baseline, 12-wk |
|  |  |  |  | Sham tDCS + PTc: 10 | MoCA:  24.20 (3.45) | 73.1(4.6) | 5(5) | Sham tDCS (only delivered during the initial 30s and ramped down to 0 mA to replicate the physical sensation) combined with Tai Chi | | 3 per wk, Sham tDCS: 20 min  TC: 40 min |  |  |
| Lin et al. (2020) | Taiwan | Community | / | Multimodal: 74 | / | 73.3(7.7) | 23(44) | Multimodal intervention: (BPSFAP intervention) include physical activities of moderate intensity (including walking and stretching exercise), cognitive stimulation activities (including Sudoku, crossword puzzles, jigsaw puzzles, calculations, maze walking, Chinese ring puzzles, and reading), and social interaction activities (chess playing, painting, handicrafts, checker playing, and poker playing). After the training program ended in week 6, the researcher discussed with each participant to gather feedback on practical activities recommended and their willingness to practice at home.  In Phase 2, researchers will phone participants once a week to follow up with their progress through their Activity Implementation Self-Inspection Checklist and help with problems that participants encountered. | | Once per wk, 90 min in phase 1 | 6 mo | Baseline, 6-mo, 12-mo follow-up |
|  |  |  |  | Active Control (HE): 67 | / | 68.5(7.9) | 18(56) | HE Control: An MCI HE leaflet was provided after the first test | | Once only |  |  |
| Liu et al. (2023) | China | Clinic | / | Multimodal: 86 | / | 69.7(6.0) | 22(64) | Multimodal Intervention: Mindfulness meditation (5 min at the beginning of all sessions led by a trained research assistant, and is required to be practiced as homework), CT (two sessions on educational information of age-related cognitive changes, eleven sessions on mnemonic strategies, four sessions on how to use smartphones, and three sessions on playing games involving the use of executive functioning, attention, and memory), physical exercise (two group sessions and daily homework including aerobic exercise, strength, and balance training), and nutrition counseling (one group session of educational lecture and two individual visits tailored for each participant, aiming to facilitate healthy dietary habits and risk management of cardiovascular diseases). | | Once per wk, around 60 min | 9 m | Baseline, 9-mo, 21-mo follow-up |
|  |  |  |  | Inactive Control: 106 | / | 73.1(5.7) | 36(70) | Inactive Control: No intervention, usual care | | / |  |  |
| Maffei et al. (2017) | Italy | Community | aMCI & naMCI: ECADWG | Multimodal: 55 | / | 74.0(4.8) | 26(29) | Multimodal Intervention: The multimodal intervention comprises of combined physical-cognitive training and music therapy. The physical-cognitive training involves aerobic exercises and an 8-cycle program targeting different cognitive abilities. These abilities include attention, memory, imagination, orientation, verbal skills, emotional memory, and logical thinking. Music therapy involves both listening activities and active participation in singing, playing musical instruments, and rhythmic movements | | PT: 2 per d, 3d per wk, 60mins;  CT: 1 per wk, 60min;  Music therapy: 1h per wk  / | 7 mo | Baseline, 7-mo, 14-mo follow-up |
|  |  |  |  | Inactive Control: 58 | / | 74.9(4.4) | 32(26) | Inactive Control: No intervention | |  |  |  |
| Martin et al. (2019) | Australia | / | sd-aMCI & md-aMCI: Albert et.al, BRANS, WTAR,  BADL | Multimodal: 33 | / | 71.8(6.4) | 13(20) | Multimodal Intervention: CCT using COGPACK, a program that targets working memory, processing speed, and attention combined with tDCS (Placed overlying the left dorsolateral prefrontal cortex, along with a ramp-up of 30s to 2mA) during the cognitive training task. | | 3 per wk, 45 to 60 min | 5 wk | Baseline, 5-wk, 3-mo follow-up |
|  |  |  |  | Control: 35 | / | 71.6(6.4) | 10(25) | Control: Sham tDCS (Initial ramp-up of 30s to 1mA for 1 min then ramp down to 0 mA in 30s to replicate the physical sensation) combined with CCT using CoGPACK. | | 3 per wk, 45 to 60 min |  |  |
| Montero-Odasso et al. (2023) | Canada | Community | Albert et al. | Multimodal:  34 | MMSE: 27.00 (2.20) | 73.1(6.1) | 13(21) | Multimodal Intervention: PT (aerobic and resistance exercise) for 60 min, CT (Neuropeak, a program that targets working memory and attention) for 30 min, a capsule of vitamin D (10,000 IU) | | 3 per wk, 90 min | 20 wk | Baseline, 6-mo, 12-mo follow up |
|  |  |  |  | PT + CT + NTc: 35 | MMSE: 27.40 (2.10) | 72.4(7.3) | 19(16) | PT + CT + NTc (placebo) | |  |  |  |
|  |  |  |  | PT + CTc + NT: 37 | MMSE: 26.30 (2.30) | 73.1(7.6) | 17(20) | PT + NT + CTc (sham cognitive training, touristic search and video watching) | |  |  |  |
|  |  |  |  | PT + CTc + NTc: 35 | MMSE: 26.90 (2.40) | 73.1(5.9) | 23(12) | PT + CTc + NTc | |  |  |  |
|  |  |  |  | Control: 34 | MMSE: 27.00 (2.20) | 73.8(6.2) | 17(17) | Control: Stretching, balance, and toning exercises. | |  |  |  |
| Parial et al. (2022) | Philippines | Community | Petersen et.al  2004 | Multimodal: 30 | MoCA:  20.67 (1.88) | 63.3(4.5) | 6(24) | Multimodal Intervention: Zumba Gold dancing for 40 min following a series of integrated cognitive tasks targeting different domains: executive function (forward and backward serial counting, perceptual-motor ability (doing arm-clock positions based on prompts), memory (forward and backward recall of word/number series), and complex attention (forward and backward spelling). Participants performed each task as a group and then transitioned from one person to another (e.g., serial counting) or identifying members (ID number that was preassigned) to perform the activities. The use of calling ID numbers aimed to further stimulate attention. | | 3 per wk, 60 min | 12 wk | Baseline, 12-wk, 18-wk follow-up |
|  |  |  |  | HE: 30 | MoCA:  20.20 (2.22) | 64.3(5.9) | 8(22) | HE: Health education that emphasized the importance of physical activities and lifestyle factors on dementia risk reduction was provided. Participants in the HE control group were instructed to perform moderate physical or leisure activities for 60 min, 3 times per week, without joining any structured group exercise program. | | 3 per wk, 60 min |  |  |
| Park et al. (2019) | Korea | Clinic | / | Multimodal: 25 | MMSE:  24.60 (2.60) | 70.6(6.5) | 8(17) | Multimodal Intervention: Participants started with a 10 min warm-up, followed by 10 min of stretching, 20 min of aerobic exercise (stair stepping, endurance walking and stair climbing, and walking on an agility ladder), 10 min of balance exercise, and 30 min of dual-task training delivered through COGNICISE (performing a cognitive task such as calculation while performing aerobic exercises), 20 min of promoting daily physical activity education, and 10 min of cooldown. | | Once per wk, 110 min | 24 wk | Baseline, 3-mo, 24-wk |
|  |  |  |  | Inactive Control: 24 | MMSEL 25.70 (3.10) | 72.8(5.4) | 17(7) | Inactive Control: No intervention, live as usual | | / |  |  |
| Phoemsapthawee et al. (2022) | Thailand | Community | aMCI: Petersen et.al 2014 | Multimodal: 20 | MMSE:  20.10 (3.00) | 73.2(4.9) | 4(16) | Multimodal Intervention: Physical exercise with 30 min of muscle strengthening, 40 min of aerobic exercise, postural balance training, and dual-task training (memorize a step pattern in consecutive square segments and step as quickly and accurately as possible while walking), and cool down for 5 min. 500 mg of Gotu kola supplements were provided which participants were advised to take twice a day. | | 3 per wk, 80 min. Supplement: twice a day | 12 wk | Baseline, 12-wk, 18-wk follow-up |
|  |  |  |  | PTc: 20 | MMSE:  20.60 (2.10) | 73.2(4.9) | 3(17) | PTc: Physical exercise only | | 3 per wk, 80 min |  |  |
|  |  |  |  | Placebo Control: 18 | MMSE:  21.20 (2.50) | 73.7(8.3) | 3(15) | Placebo Control: Only placebo Gotu kola supplement was provided | | Twice per day |  |  |
| Rezola-Pardo et al. (2019) | Spain | Community | / | Multimodal: 42 | MoCA:  13.60 (5.70) | 85.3(7.1) | 15(28) | Multimodal Intervention: Participants were to perform moderate strength and balance exercises. In addition, participants in the multimodal intervention group were to participate in dual-task training. All cognitive tasks were tailored individually targeting attention, executive functions, and semantic memory. The challenge will gradually progress along with the complexity of the physical exercise and cognitive task. | | 2 per wk, around 60 min | 3 mo | Baseline, 3-mo |
|  |  |  |  | PTc: 43 | MoCA: 12.00 (4.50) | 84.9(6.7) | 13(29) | PTc: Participants were to perform moderate strength and balance exercises. | | 2 per wk, around 60 min |  |  |
| Senczyszyn et al. (2023) | Poland | Community | Petersen et al. | rTMS + CCT: 11 | MoCA:  23.82 (2.71) | 70.6(3.14) | 6(5) | Multimodal Intervention: rTMS (10 Hz) and CCT (RehaCom, computerized CT targeting attention, memory, visuospatial processing, and executive functions) | | 5 per wk | 2 wk | Baseline, 2-wk |
|  |  |  |  | rTMS: 11 | MoCA: 24.82 (1.17) | 70.7(4.5) | 7(4) | rTMS only | |  |  |  |
|  |  |  |  | Sham Control: 9 | MoCA: 24.56 (1.74) | 71.6(5.7) | 8(1) | rTMS delivered by a sham coil | |  |  |  |
| Shimada et al. (2018) | Japan | Community | aMCI & naMCI: NCGG-FAT | Multimodal: 154 | MMSE:  26.60 (1.80) | 71.6(5.0) | 77(77) | Multimodal Intervention: This combined activity program includes a 10 min warm-up and stretching, 20 min of muscle strength exercises and postural balance training, and 25 min of dual-task training using COGNICIZE, a 5 min rest will then be given followed by a 25 min of aerobic exercise and 5 min of cooling down. | | Once per wk, 90 min | 40 wk | Baseline, 40-wk |
|  |  |  |  | HE: 154 | MMSE:  26.80 (1.80) | 71.6(4.9) | 77(77) | HE: Health education will be provided regarding topics such as aging, nutrition, oral care, frailty, and urinary incontinence. Pamphlets about the topics will be mailed thrice. | | 3 over the study period, 90 min |  |  |
| Shimizu et al. (2018) | Japan | Community | Petersen et.al 1999 &2004 | Multimodal: 34 | FAB:  15.00 (2.00) | 74.9(4.3) | 6(28) | Multimodal Intervention: Participants were to exercise, sing loudly, using the Naruko clapper, and perform synchronizing exercise movements with those of the instructors for 60 sec | | Once a wk, 65 min | 12 wk | Baseline, 12-wk, 18-wk follow-up |
|  |  |  |  | PTc: 11 | FAB:  15.10 (2.00) | 73.3(7.3) | 1(10) | PTc: Perform the same task as the multimodal intervention group but without music or using the Naruko clapper | |  |  |  |
| Straubmeier et al. (2017) | Germany | Community | / | Multimodal: 208 | / | 81.5(7.5) | 81(127) | Multimodal Intervention: MAKS therapy, consists of 4 major components, in which participants will enter an approximately 10 min social warm-up, followed by a sensorimotor activation (general mobility, gross and fine motor skills, balance, and sensory perception) training through games and sports lasted around 30 min, and cognitive activation training through projector or pen-and-paper exercises for around 30 min after a short break. Finally, participants will then enter the activation of activities of daily living for 40 min, which helps practice gross and fine motor skills, mobility and procedural memory. | 1 to 5 per wk, around 2 hr | | 6 mo | Baseline, 6-mo |
|  |  |  |  | Inactive Control: 154 | / | 81.1(7.5) | 60(94) | Inactive Control: No intervention, usual care. | | / | / |  |
| Stuerz et al. (2022) | Austria | Community | / | Multimodal: 26 | MMSE:  24.80 (3.50) | 87.0(7.0) | 1(25) | Multimodal Intervention: Relaxation training consists of a progressive muscle relaxation technique that helps reduce stress and muscular tension with music for 3 weeks. After the first 3 weeks, CT that targets attention, information processing, and memory will introduce to the participants several cognitive tasks in paper-and-pencil-based training based on the COGPACK program along with short RT. | | 2 per wk, 30 to 35 min | 6 wk | Baseline, 3-wk, 6-wk |
|  |  |  |  | Reverse Sequential Control: 24 | MMSE:  26.30 (2.70) | 87.0(5.0) | 0(24) | Multimodal Intervention Control: CT for 3 weeks, followed by relaxation training with short CT | | 2 per wk, 30 to 35 min |  |  |
| Styliadis et al. (2015) | Greece | Clinic | Petersen et.al  2004 | Multimodal: 14 | MMSE:  25.85 (2.09) | 71.2(4.5) | 5(9) | Multimodal Intervention: Long Lasting Memories project (LLM), a combined training with PT (aerobics, strength, balance, and flexibility training) delivered through FitForAll using Nintendo Wii and its supporting hardware, and CT (auditory processing and working memory training) using the Greek adaption of the Brain Fitness software. | | PT: 5 per wk, 60 min  CT: 3 to 5 per wk, 60 min | 8 wk | Baseline, 8-wk |
|  |  |  |  | PTc: 14 | MMSE:  26.21 (2.33) | 70.4(6.6) | 5(9) | PTc: PT only | | 5 per wk, 60 min |  |  |
|  |  |  |  | CTc: 14 | MMSE:  25.14 (3.22) | 72.7(6.6) | 5(9) | CTc: CT only. | | 3 to 5 per wk, 60 min |  |  |
|  |  |  |  | HE: 14 | MMSE:  26.21 (1.97) | 71.1(4.4) | 5(9) | Watching documentaries on YouTube. | | 3 to 5 per wk, 60 min |  |  |
|  |  |  |  | Inactive Control: 14 | MMSE:  25.00 (1.77) | 67.6(4.0) | 5(9) | Inactive Control: No intervention. | | / |  |  |
| Tao et al. (2023) | China | Community | / | Multimodal: 51 | MMSE: 24.33 (1.79) | 73.4(7.8) | 16(35) | Multimodal Intervention: Combined PT (five sense exercise, brain exercises and breathing exercise) and CT (targeting attention, memory, and executive function). | | Once per 2 wk, 60 to 90 min | 3 mo | Baseline, 3-mo, 6-mo follow up |
|  |  |  |  | HE: 52 | MMSE: 24.00 (1.72) | 74.9(6.6) | 20(32) | HE only. | |  |  |  |
| Xu et al. (2020) | Hong Kong | Community | / | Multimodal: 6 | ADAS-Cog:  12.44 (3.25) | 70.7(4.2) | 2(4) | Multimodal Intervention: Combined mind-body physical exercise, cognitive training, and risk factor modification (CPR) consist of a 30 min 24-form simplified TC taught by a TC master or trained physical therapists, CT using Rummikub, and the Risk Factor Modification (RFM) intervention consisting of two part: nutritionary intervention (personalized nutrition, discussion and exercises to promote lifestyle changes) and management of metabolic and vascular risk (providing health-related information on the importance of reducing risk factors) through motivational interviewing using FRAMES (feedback, responsibility, advice, menu, empathy, and self-efficacy). | CT: 3 per wk, 60 min  RFM: 3 over the study period  TC: at least 3 per wk, 30 min | | 6 mo | Baseline, 6-mo |
|  |  |  |  | Nutrition + HE: 7 | ADAS-Cog:  13.40 (4.72) | 76.4(4.5) | 2(5) | Nutrition + HE: RFM only. | | 3 over the study period |  |  |
|  |  |  |  | Inactive Control: 6 | ADAS-Cog:  13.33 (2.76) | 74.5(6.0) | 1(5) | Inactive Control: HA would be given in the form of booklets would be provided for the participants. | | / |  |  |
| Xu et al. (2023) | China | Community | / | Multimodal: 44 | / | 59.0(8.8) | 12(32) | Multimodal Intervention: Combined PT (24-form Tai Chi) for 60 min and tDCS (2 mA placed over F4 (EEG 10-20 standard system) and Fp1 (EEG 10-20 standard system) for the first 20 min. | | 3 per wk, 60 min | 12 wk | Baseline, 12-wk |
|  |  |  |  | Multimodal: 44 | / | 63.0(12.8) | 21(23) | Multimodal Intervention: PT (walking) for 60 min and tDCS (2 mA placed over F4 (EEG 10-20 standard system) and Fp1 (EEG 10-20 standard system) for the first 20 min. | |  |  |  |
|  |  |  |  | PT + Sham tDCS: 49 | / | 61.0(8.5) | 19(30) | PT + Sham tDCS: Combined PT (24-form Tai Chi) for 60 min and sham tDCS. | |  |  |  |
|  |  |  |  | PT + Sham tDCS: 43 | / | 58.0(8.0) | 17(26) | PT + Sham tDCS: Combined PT (walking) and sham tDCS. | |  |  |  |
| Yang et al. (2022) | China | Community | Petersen et.al 1997 | Multimodal: 55 | MoCA:  21.00 (1.38) | 70.7(5.4) | 27(28) | Multimodal intervention: Combined nutritionary intervention (meeting with community dietitians face to face to provide information and support on lifestyle improvement, and building healthy nutrition), PT (individualized progressive muscle strength training and aerobic exercise), and CCT that targeted memory but also involved attention, executive function, visual-spatial ability, language, abstraction, calculation, orientation, and other cognitive domains. | | Dietary intervention: once every 3-4 wk (6 sessions in total)  PT: 1/wk at the first month and twice per wk for the remaining months  CT: once per wk | 6 mo | Baseline, 6-mo, 7-mo & 9-mo & 12-mo follow-up |
|  |  |  |  | HE: 57 | MoCA:  21.48 (1.42) | 70.5(6.5) | 26(31) | HE: Usual care and three HE classes across the study period. | | 3 class, 45 min |  |  |
| Note. ACE-R = Addenbrooke’s Cognitive Examination-Revised; ADAS-Cog = Alzheimer's disease assessment scale-Cognitive subscale; aMCI = Amnestic Mild Cognitive Impairment; BADL = Bayer-Instrumental Activities of Daily Living; BRANS = Repeatable Battery for the Assessment of Neuropsychological Status; CCT = Computerized Cognitive Training; CCTc = Computerized Cognitive Training Control; CT = Cognitive Training; CTc = Cognitive Training Control; DASH = Dietary Approaches to Stop Hypertension; EA = Electroacuputure treatment; ECADWG = The European Consortium on Alzheimer’s Disease Working Group on MCI; HA = Health Advice; HE = Health Education; ICD-9-CM = International Classification of Diseases, 9th Revision, Clinical Modification; mdMCI = Multi-domain Mild Cognitive Impairment; MIP = Memory Intervention Program; MMSE = Mini-Mental State Examination; mo = Month; MoCA = The Montreal Cognitive Assessment; naMCI = non-amnestic Mild Cognitive Impairment; NCGG-FAT = National Center for Geriatrics and Gerontology-Functional Assessment Tool; FAB = Frontal Assessment Battery; PRT = Progressive Resistance Training; PT = Physical Training; PTc = Physical Training Control; RT = Reminiscence Therapy; sdMCI = Single-domain Mild Cognitive Impairment; TC = Tai Chi; TTC = Taoist Tai Chi; wk = week; WTAR = Wechsler Test of Adult Reading.  ^a^Reflects the number and percentage of participants for the whole sample. | | | | | | | | | | | | |
