# Supplementary material for: Nonpharmacological Multimodal Interventions for Cognitive Functions in Older Adults With Mild Cognitive Impairment: Scoping Review
Source: JMIR Aging. 2025 May 12;8:e70291. doi: 10.2196/70291 (PMC12107202; doi:10.2196/70291)
Supplement: Multimedia Appendix 3 [file aging_v8i1e70291_app3.docx]

| **Appendix Table 3.** Computerized training program used in the included studies. | | | | | |
| --- | --- | --- | --- | --- | --- |
| Name of Program | Task Involved | Other electronic usages | Progression | Duration | Outcome |
| AKL-T01 | Visuomotor/sensorimotor task perceptual discrimination task (Jones et al., 2023) | iPad as a steering wheel | Difficulty increases progressively | Participants completed 5 ‘missions’ per day, which lasted approximately 20-min in total. | ATT: (Cohen *d*=.94, ***P*=.047**)  ME: (η^2^=.05, *P*=.346) |
| Brain Fitness | Auditory stimuli targeting Auditory processing and working memory (Styliadis et al., 2015) | / | Self-paced levels of difficulty | 3-5 times per week; 1 hour per day for 8 weeks; 15 min per exercise. | No significant changes across all groups. |
| CogniPlus | Subprogrammes targeting attention, working memory, long-term memory, executive function, planning of daily activities, and visuomotor ability (Hagovska et al., 2016) | / | Difficulty increases progressively | 2 sessions per week; 30 minutes per session; The program consists of 20 sessions in total, and each session consists of three programs to ensure all cognitive functions can be trained. | ATT (SCWT: η^2^=.0001, *P*=.966)  GC (ACE: Cohen *d*=.71, ***P*=.002**, MMSE)  ME (ACE: Cohen *d*=.64, ***P*=.007**; AVLT: η^2^=.173, ***P=*.0001**)  PS (DRT-II: η^2^=.033, *P=*.106)  VF (Cohen *d*=.73, ***P*=.001**) |
| COGPACK | Visual and verbal explicit memory tasks targeting memory, executive function, attention, and processing speed (Singh et al., 2014) | Touch screen | Difficulty increases progressively | 2-3 times per week; 75 min per session. | No significant differences compared (*P*>.05) |
| FitForAll | Physical exercises including flexibility, balance, strength, and endurance through aerobic training (Styliadis et al., 2015) | Nintendo Wii, remote, and balance board | Adjusted for older adults' capacity. | 5 sessions per week; 1 hour per day for 8 weeks; The program consists of 20 min of aerobic exercises, 8-10 min of resistance exercises, 10 min of flexibility exercises, and a set of balance-targeted exercises. | No significant changes across all groups. |
| Neuron Up | Memory and attention tasks targeting executive function, memory, and attention (Gonzalez et al., 2021) | / | Difficulty increases progressively | 3 sessions per week; 30 min per session for 3 weeks; The program consists of 9 sessions in total. | ATT: TMT-A (*F*=.64, *P*=.62)  GC: MoCA (*F*=.34, *P*=.85)  ME: DST (F=1.99, *P*=.09); RBMT-3 (*F*=.13, *P=*.96) |
| Neuropeak | Visuomotor tasks targeting working memory and attention (Montero-Odasso et al., 2023) | iPad | Difficulty increases progressively | 3 session per week, 30 min per session for 20 weeks; The program consists of 60 sessions in total. | GC: ADAS-Cog (mean difference = -2.64, ***P=*.005,** d= 0.71)  EF (ADAS-Cog Plus variant: no significant improvement (*P>.05*) |
| RehaCom | Cognitive tasks targeting executive function (Kim & Park, 2023) | / | Difficulty increases progressively | 2 sessions per week; 45 min per session for 8 weeks; The program consists of 16 sessions in total. | EF: (EFPT-K: η^2^=.132, ***P*<.01**; FAB: η^2^=.305, ***P*<.001**) |
|  | Cognitive task targeting attention, memory, and executive function (Kim et al., 2020) | / | / | 3 sessions per week; 30 min per session for 8 weeks; The program consists of 24 sessions in total. | GC: (ADAS-Cog-K: Z=-.38, *P*=.703; MoCA-K: Z=-.72, *P*=.47) |
|  | Cognitive tasks targeting attention, memory, visuospatial processing, and executive function (Senczyszyn et al., 2023) | / | Difficulty increases progressively | 5 sessions per week; 30 min per session for 2 weeks; The program consists of 10 sessions in total. | GC: CANTAB (Swms6: η^2^=.042, *P>*.05; Palta4: η^2^=.019, ***P*=.027**; Prmpci: η^2^=.091, ***P*=.023**)  VF (η^2^=.042, *P>*.05) |
| Not Specified* | Cognitive tasks targeting memory, attention, executive function, visual-spatial ability, language, math ability, orientation, and other cognitive domains (Yang et al., 2022) | / | Difficulty increases progressively | 1 session per week, 60-90 min per session for 24 weeks. The program consists of 24 sessions in total. | GC: Group X Time Interaction (***P*<.001**) |
|  | Cognitive game targeting executive function, attention, and memory (Lau et al., 2024) | Nintendo Switch | Difficulty increases progressively | 3 sessions per week, 40 min per session for 5 weeks. The program consists of 15 sessions in total. | GC: MoCA (η^2^=.05, *P*=.35)  EF: TMT-A (η^2^= .18, *P*=.06); TMT-B (η^2^= .12, *P*=.13);  Tower of London (η^2^=.03, *P*=.42);  ME: N-back (1-back: η^2^=.17, *P*=.07; 2-back: η^2^=.16, *P*=.07); CVVLT (verbal memory: η^2^=.12, *P*=.07; delayed recall: η^2^=.02, *P*=.59) |
| *Note.* Bold indicates statistically significant. ACE = Addenbrooke’s cognitive examination; ADAS-Cog = Alzheimer’s disease assessment scale-cognitive; ADAS-Cog-K = Alzheimer’s disease assessment scale-cognitive, Korean version; ATT = attention; AVLT = auditory verbal learning test; CANTAB = Cambridge neuropsychological test automated battery; CVVLT = Chinese version of the verbal learning test; DRT-II = ; DST = digit span test; EF = executive function; EFPT = executive function performance test; FAB = frontal assessment battery; GC = global cognition; MMSE = mini-mental state examination; MoCA = Montreal cognitive assessment; ME = memory; PALT = paired associative learning test; PS = processing speed; RBMT-3 = riverheads behavioral memory test; SCWT = Stroop color-word test; TMT-A = trial making test-A; TMT-B = trail making test-B;VF = verbal fluency | | | | | |
